# Molecular Mechanisms Linking Genes and Vitamins of the Complex B Related to One-Carbon Metabolism in Breast Cancer: An In Silico Functional Database Study

**DOI:** 10.3390/ijms25158175

**Published:** 2024-07-26

**Authors:** José María Gálvez-Navas, Esther Molina-Montes, Miguel Rodríguez-Barranco, MCarmen Ramírez-Tortosa, Ángel Gil, María-José Sánchez

**Affiliations:** 1 Centro de Investigación Biomédica en Red de Epidemiología y Salud Pública (CIBERESP), 28029 Madrid, Spain; jmaria.galvez.easp@juntadeandalucia.es (J.M.G.-N.); mariajose.sanchez.easp@juntadeandalucia.es (M.-J.S.); 2 Cancer Registry of Granada, Andalusian School of Public Health, Campus Universitario de Cartuja, Cuesta del Observatorio 4, 18011 Granada, Spain; 3 Instituto de Investigación Biosanitaria ibs. GRANADA, Av. de Madrid, 18012 Granada, Spain; agil@ugr.es; 4 Ph.D. Program in Nutrition and Food Sciences, International Postgraduate School, University of Granada, Av. de Madrid 13, 18012 Granada, Spain; 5 Department of Nutrition and Food Sciences, Faculty of Pharmacy, University of Granada, Campus Universitario de Cartuja, 18011 Granada, Spain; 6 Department of Biochemistry and Molecular Biology II, Faculty of Pharmacy, University of Granada, Campus Universitario de Cartuja, 18011 Granada, Spain; mramirez@ugr.es; 7 Centro de Investigación Biomédica en Red de la Fisiopatología de la Obesidad y Nutrición (CIBEROBN), 28029 Madrid, Spain

**Keywords:** one-carbon metabolism, breast cancer, genes, single nucleotide polymorphisms, B-complex vitamins, computational biology

## Abstract

Carcinogenesis is closely related to the expression, maintenance, and stability of DNA. These processes are regulated by one-carbon metabolism (1CM), which involves several vitamins of the complex B (folate, B2, B6, and B12), whereas alcohol disrupts the cycle due to the inhibition of folate activity. The relationship between nutrients related to 1CM (all aforementioned vitamins and alcohol) in breast cancer has been reviewed. The interplay of genes related to 1CM was also analyzed. Single nucleotide polymorphisms located in those genes were selected by considering the minor allele frequency in the Caucasian population and the linkage disequilibrium. These genes were used to perform several in silico functional analyses (considering corrected *p*-values < 0.05 as statistically significant) using various tools (FUMA, ShinyGO, and REVIGO) and databases such as the Kyoto Encyclopedia of Genes and Genomes (KEGG) and GeneOntology (GO). The results of this study showed that intake of 1CM-related B-complex vitamins is key to preventing breast cancer development and survival. Also, the genes involved in 1CM are overexpressed in mammary breast tissue and participate in a wide variety of biological phenomena related to cancer. Moreover, these genes are involved in alterations that give rise to several types of neoplasms, including breast cancer. Thus, this study supports the role of one-carbon metabolism B-complex vitamins and genes in breast cancer; the interaction between both should be addressed in future studies.

## 1. Introduction

According to the International Agency for Research on Cancer, breast cancer is the leading neoplasm in incidence and mortality in women worldwide. In 2022, there were 2.3 million women diagnosed with this disease [1]. Current evidence indicates the existence of a wide range of well-known risk factors for the disease, such as alcohol intake, obesity, substitutive hormonal treatment usage, physical inactivity, no breastfeeding, breast density, family history of breast cancer, and genetics. Furthermore, mutations in *BRCA1* and *BRCA2* genes are responsible for 5–10% of breast cancer cases [2,3]. However, more than 50% of new breast cancer cases do not present any other risk factor than age [4].

**Figure 1:**
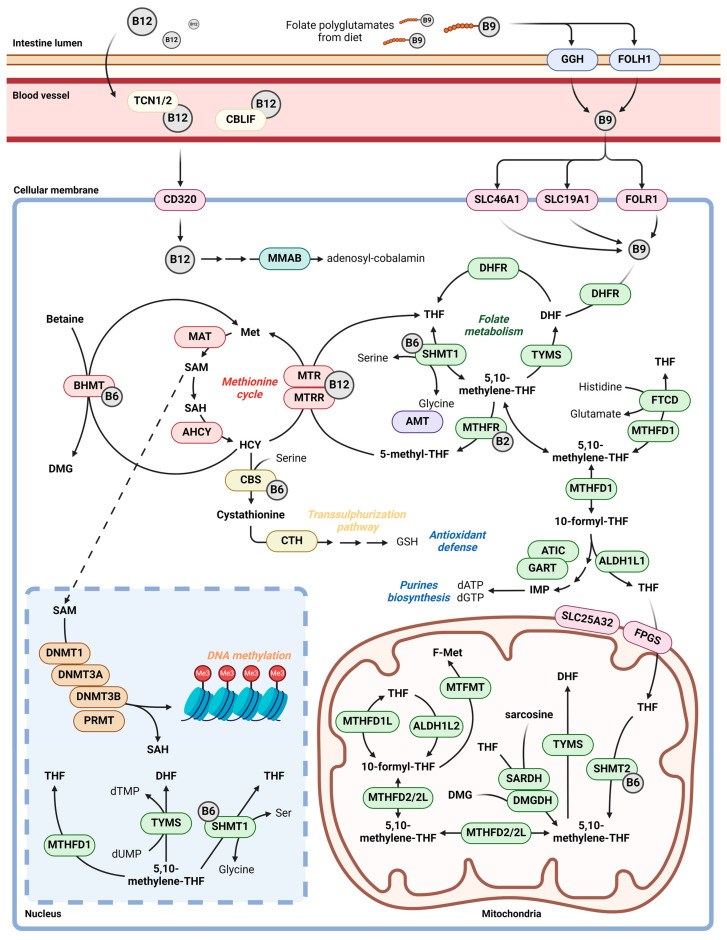
One-carbon metabolism pathway. DHF: dihydrofolate, THF: tetrahydrofolate, IMP: inosine monophosphate, Met: methionine, SAM: S-adenosylmethionine, SAH: S-adenosylhomocysteine, HCY: homocysteine, DMG: dimethylglycine, dATP: deoxyadenosine triphosphate, dGTP: deoxyguanosine triphosphate, dTMP: deoxythymidine monophosphate, dUMP: deoxyuridine monophosphate, Ser: serine.

Carcinogenesis is a complex cellular phenomenon related to DNA stability and expression, driven by the dysregulation of various cellular pathways due to the presence of genetic mutations and the erratic activity of DNA-repair enzymes. In addition, epigenetic mechanisms such as DNA methylation or histone modifications may induce cell phenotypic changes, either independently or through genetic mutations [5,6]. DNA methylation is regulated by one-carbon metabolism (1CM), which is a complex network of biochemical reactions that involve the folate metabolism, the methionine cycle, and the trans-sulphuration pathway. Folate metabolism occurs in the liver, while methionine cycle, trans-sulphuration, and substrate methylation take place in the target tissue. Apart from folate, which initiates the cycle, vitamins B2, B6, and B12 participate in 1CM as enzymatic cofactors. Additionally, alcohol is a common dietary component related to this pathway due to the inhibition of folate absorption and metabolism (Figure 1) [7,8]. The 1CM regulates the cellular function throughout the one-carbon moieties (methenyl, formyl, and methyl groups), which are required for molecular biosynthesis, regulation of nucleotide pools, epigenetic control of gene expression, and redox defense. Thus, 1CM is involved in several cellular mechanisms, such as growth and proliferation [9]. Also, the 1CM pathway presents a dual role in carcinogenesis. On the one hand, it maintains cellular stability in the previous stages of its initiation. On the other hand, once the carcinogenic process has been triggered, 1CM acquires a central role in the tumoral cell due to its involvement in cell proliferation [10]. Thus, nutrients, genes, and single nucleotide polymorphisms (SNPs) related to 1CM may influence breast carcinogenesis. Concerning breast cancer, several studies have provided evidence about the relationship between the 1CM participating enzymes or involved nutrients and the disease throughout epidemiological studies and in vivo and in vitro models. The dietary methyl group (folate, choline, betaine, and methionine) intake role in breast cancer has been assessed within the European Prospective Investigation into Cancer and Nutrition (EPIC) cohort, enrolling 318,686 women (13,320 malignant breast cancer cases). Results showed a potential U-shaped relationship between dietary folate intake and overall breast cancer risk [11]. In addition, the relationship between folate and triple-negative breast cancer subtype (TNBC) has been assessed in vitro and in vivo. Folate restriction promotes changes in cells, decreasing their migratory capacity and invasiveness, which is associated with depleted metabolic plasticity. These effects were higher in murine models with mitochondrial disfunction, which leads to an increased dependence on 1CM for tumoral cells [12,13]. Moreover, it has been noted that loss of ALDH1L2, a 1CM-related enzyme, drives an increased migration in breast cancer cells and enhances metastasis in vivo [14]. Indeed, different metabolomic profiles have been observed between invasive ductal carcinoma and adjacent tissue, where 1CM-involved metabolites presented a pivotal role [15]. To provide evidence of their role in this disease, it is possible to make use of biological databases on genes and molecular, metabolic, and cellular pathways linking genes and their related SNPs with diseases. These biological databases are, therefore, valuable tools for conducting in silico functional analyses from different sources, providing molecular mechanisms and facilitating the interpretation and visualization of the results.

Thus, this study aimed to interrogate some biological databases on the potential associations between the genes related to 1CM and cancer, focusing on breast cancer, to explore the possible underlying mechanisms between them. Furthermore, we have revised the association of the water-soluble B group vitamins involved in 1CM (folate, B2, B6, and B12) and alcohol with breast cancer.

## 2. Results

### 2.1. Relationship between Dietary Components Related to One-Carbon Metabolism (Vitamins B2, B6, B9, B12, and Alcohol) and Breast Cancer

The B group vitamins (B1, B2, B3, B5, B7, B9, and B12) are involved in a wide range of essential processes in cellular activity maintenance. Several of these B vitamins are involved in 1CM, either by initiating the cycle, as in the case of folate, or by participating as enzyme cofactors. Additionally, alcohol involvement in this pathway lies in its inhibition of folate activity [7,8,9,10]. In the text, alcohol and ethanol will be treated as synonyms. While several studies support that those 1CM nutrients are meaningful for breast cancer prevention, some inconsistencies remain (Table S1).

#### 2.1.1. Vitamin B2 (Riboflavin)

Riboflavin, vitamin B2, or lactoflavin is part of the water-soluble vitamin group. It has two coenzyme derivatives responsible for its biological activity: flavin mononucleotide (FMN) and flavin adenine dinucleotide (FAD) [16]. Vitamin B2 is found in foods of both animal (organ meats, eggs, and fish) and green vegetable origins in its co-enzymatic form, bound to apoenzymes. However, the free form of riboflavin is found in milk, which is the main food source [17].

Riboflavin plays a key role in the metabolism of carbohydrates, lipids, and proteins. FMN and FAD are closely related to flavoenzymes, which are involved in oxidation–reduction reactions and enable electron transport through the transformation of the isoalloxazine ring [17]. The functional versatility of FMN and FAD means that they are involved in a wide variety of cellular processes, such as ATP generation through the mitochondrial electron transport chain or cellular antioxidant defense. They are also involved in 1CM as coenzymes of the enzyme methylenetetrahydrofolate reductase (MTHFR), a key enzyme in the initiation of methionine metabolism. Oxidative stress and DNA methylation are processes closely linked to cell proliferation and, thus, to cancer [16,18].

Riboflavin participates in 1CM as an enzyme cofactor of the enzyme MTHFR. Therefore, dietary intake and serum levels of riboflavin could influence the development of breast cancer. Concerning the relationship between plasma levels of this vitamin and breast cancer risk, the meta-analysis conducted by Zeng et al. (2020) (27 prospective case–control and cohort studies) concluded that there is no relationship between blood levels of riboflavin and breast cancer risk [19]. On the other hand, dietary intake of riboflavin has been linked to the development of breast cancer in the study conducted by Hatami et al. (2020) (n = 151 cases/154 controls), showing that higher intake of B2, as measured by validated food frequency questionnaires, is associated with a lower risk of the disease [20]. However, the Canadian Study of Diet, Lifestyle, and Health cohort study (n = 922 cases/3088 controls) showed no significant association between riboflavin intake and different types of cancer, including breast cancer [21]. The association between high vitamin B2 intake and a lower risk of developing breast cancer is supported by two meta-analyses, both of which agree on this issue. The one conducted by Yu et al. (2017) (n = 12,268 cases/194,530 controls) showed a weak association, while the one conducted by Zeng et al. (2020) (n = 49,707 cases/1,274,060 controls) showed a stronger association [19,22].

#### 2.1.2. Vitamin B6 (Pyridoxine, Pyridoxal, and Pyridoxamine)

The term vitamin B6 groups together three water-soluble pyridine derivatives: pyridoxine, pyridoxamine, and pyridoxal, which are metabolically interconvertible. Vitamin B6 is abundant in foods in all forms, especially liver, legumes, nuts, and bananas. Pyridoxine and pyridoxamine are found mainly in vegetables, while pyridoxal predominates in animal products [17]. The active form of vitamin B6 is pyridoxal phosphate (PLP), which intervenes as a coenzyme in multiple key reactions of amino acid metabolism. The action of PLP has three possible effects on amino acids, acting primarily as transaminases [18]. Vitamin B6 deficiency has been linked to an increased risk of cancer due to PLP’s protective role against DNA damage, among other mechanisms [23]. B6 is also involved in 1CM as an enzymatic factor in serine hydroxy-methyltransferase 1 and cystathionine β-synthase, both of which are involved in serine metabolism [7].

Vitamin B6 is involved as an enzyme cofactor for the enzymes serine hydroxymethyltransferase and cystathionine-β-synthase, which are involved in amino acid metabolism, a process essential for the maintenance of cell proliferation [10]. Thus, higher B6 intake is associated with a lower risk of overall breast cancer, as well as a lower risk of the ER+, PR+, or HER2 subtypes [20]. The effect of dietary intake and supplementation, as well as the combination of both, on breast cancer risk has also been evaluated. The study conducted within the prospective NutriNet-Santé cohort (N = 27,853 individuals, n = 462 incident cases of breast cancer), using 24 h recall and supplementation-specific questionnaires, indicated that both high intake and supplementation and the sum of both decrease breast cancer risk [24]. In line with these results is the meta-analysis by Zeng et al. (2020), which showed that high B6 intake reduces the risk of overall breast cancer and that of ER+/PR+ breast cancer subtypes [19]. Furthermore, vitamin B6 intake is inversely related to breast density [25]. Regarding the relationship between plasma vitamin B6 levels and breast cancer risk, the dose–response meta-analysis conducted by Wu et al. (2013) indicated that elevated levels of vitamin B6 and methionine reduce breast cancer risk, mainly in postmenopausal women [26]. However, subsequent studies have not shown a statistically significant association between plasma vitamin B6 levels and breast cancer risk [27,28]. Similarly, it has been observed that vitamin B6 may play a role in other aspects of breast cancer beyond risk. Indeed, high-dose vitamin B6 has been shown to potentiate the antitumor effect of 5-fluorouracil and folinic acid in women with advanced breast cancer [29].

#### 2.1.3. Vitamin B9 (Folate and Folic Acid)

Vitamin B9 encompasses folic acid and all its reduced derivatives. All forms share a common structure, which is that of pteroylglutamic acid or folic acid itself. There are three distinct parts: a pteridine ring, a *p*-aminobenzoic acid residue, and a glutamate residue [30]. The number of glutamate residues can vary, being found as monoglutamates, pentaglutamates, and hexaglutamates [18,31]. The pteridine ring can be partially reduced at positions 7 and 8 (dihydrofolate or DHF) or completely reduced at positions 5, 6, 7, and 8 (tetrahydrofolate or THF). THF can accept methyl units, which are attached to positions 5, 10, or both [31]. Food sources of folate are mainly of plant origin, such as chard, spinach, turnip greens, and chickpeas [18,31]. In foods, it is usually found in reduced form and bound to polyglutamates. The structure presented by folic acid is not common in nature, although it is the most stable and, therefore, the one used for its commercialization as a supplement [30]. Folate is responsible for initiating 1CM, from which all its biological functions derive [7]. Vitamin B9 is involved in serine metabolism, the synthesis of *S*-adenosylmethionine (a key molecule in transmethylation reactions), and the synthesis of purines, especially thymine [30]. Together, these mechanisms support the key role of folates in cell proliferation and, therefore, their relationship with cancer. In fact, folate is known to have a dual role in this pathology, as it can both prevent the onset of cancer and maintain tumor survival once the disease has appeared [18,31,32].

Vitamin B9 or folate initiates 1CM, regulating gene expression and maintaining DNA stability. Folate, along with methionine, homocysteine, choline, and betaine, is one of the methyl donor nutrients [7]. Due to the role played by folate in the regulation of cell proliferation, amino acid metabolism, and the synthesis of nitrogenous bases, it has been considered that a high folate intake may have a protective role against cancer [30]. On the one hand, data from food fortification in the United States show that serum folate levels, both before and after fortification, are not associated with breast cancer risk [33]. However, several studies have found that women with higher folate intakes are less likely to develop breast and ovarian cancer [21], as well as hormone receptor-positive and HER2-positive subtypes [20]. This is reinforced by the results of the meta-analysis conducted by Zeng et al. (2020) (27 case–control and cohort studies), which found that dietary folate intake is inversely associated with the development of breast cancer. Furthermore, an increase of 100 μg/day of folate through diet has been found to reduce the risk of ER-/PR- breast cancer by 7% [19]. Furthermore, dietary folate intake has been linked to other key aspects of breast cancer, such that high intake is inversely related to breast density [25]. Concerning blood folate levels, high plasma levels of B9 have been associated with an increased risk of breast cancer in women with *BRCA1* and *BRCA2* mutations [27]. However, results of the European Prospective Investigation into Cancer and Nutrition (EPIC) cohort study (n = 2492 cases/2521 controls) showed that there is no clear association between folate levels and breast cancer risk [33].

#### 2.1.4. Vitamin B12 (Cobalamin)

Cobalamin, or vitamin B12, has a complex structure in which the four pyrrole rings are arranged similarly to porphyrins, with a cobalt atom as the central nucleus. It also can bind to various ligands, giving rise to the various forms of vitamin B12. Its active forms are adenosyl-cobalamin and methyl-cobalamin [30]. This vitamin is synthesized exclusively by microorganisms; plants do not need it and, therefore, do not contain it. The source for animals is the ingestion of microorganisms or production by the intestinal microbiota [32]. Thus, the foods with the highest cobalamin content are liver, kidney, and brain, although egg yolk, sardines, salmon, clams, and oysters also have a high vitamin B12 content [30,34]. There are two metabolic reactions in which vitamin B12 is involved: the conversion of homocysteine to methionine and the conversion of L-methylmalonyl-CoA to succinyl-CoA. These biochemical functions highlight the need for cobalamin in the maintenance of the central and peripheral nervous system through the methylation of myelin, neurotransmitters, and phospholipids. It is also involved in nucleotide synthesis through its participation in 1CM [18,30]. In addition, B12 is involved in the regulation of certain immune cells, including natural killer and CD8+ T lymphocytes [18].

Due to its role in 1CM, vitamin B12 is involved in DNA methylation and proper expression, as well as nucleotide synthesis. These phenomena are critical in cancer initiation and development [18]. In fact, several reviews of the scientific literature have associated higher cobalamin levels with an elevated risk of various types of cancer, including breast, liver, and lung cancer [18,35]. Cobalamin has diverse biochemical and physiological functions due to its activity as a cofactor for the enzymes methionine synthase and methionine synthase reductase. These enzymes link folate and methionine metabolism in 1CM [8]. The scientific literature has provided contradictory results on the association between B12 intake and breast cancer. On the one hand, some studies do not support a connection between this vitamin and breast cancer disease [28,35]. However, the study by Hatami et al. (2020) demonstrated that higher vitamin B12 intake decreases the risk of breast cancer overall and of ER+, PR+, and HER2 subtypes [20]. Furthermore, like dietary intake of B6 and folate, B12 intake has been inversely related to breast density [25]. Moreover, there is more consensus on plasma cobalamin levels and disease. The dose–response meta-analysis by Wu et al. (2013) indicated that there is no relationship with breast cancer [26]. In line with these results, other studies support this lack of association [21,27].

#### 2.1.5. Alcohol

Alcohol is the most widely consumed substance in the world. Thus, its moderate consumption is taken into account in the Mediterranean diet. However, alcohol consumption has been linked to an increased risk of several chronic pathologies, including cancer. Indeed, it is the only dietary component established as a risk factor for breast cancer. Consumption of 10 g of alcohol per day has been found to increase the risk of breast cancer by 10.5% and 11.1% in postmenopausal women. In addition, occasional heavy alcohol consumption is associated with an increased risk of breast cancer compared to low–moderate and prolonged consumption. However, it should be noted that the only safe amount of alcohol consumption is 0 g/day [34,35,36,37,38,39]. The role of alcohol in cancer development is primarily attributable to two mechanisms: its ability to inhibit the action of folate, preventing the initiation of 1CM; and the cytotoxic effect of its metabolites. Alcohol metabolism involves several enzymes performing at the hepatic level that catalyze the molecule via two pathways: non-oxidative and oxidative. The latter involves the CYP2E1 enzyme, which belongs to the cytochrome P450 superfamily [37]. The oxidative pathway in alcohol metabolism is thought to lead to cell damage by reactive oxygen species and acetaldehyde. Although the production of these metabolites mainly occurs in the liver, it also takes place in breast tissue. Additionally, an accumulation and persistent concentration of acetaldehyde in breast cells compared to blood in animal models has been observed [40]. On the other hand, alcohol’s role in carcinogenesis is also related to the inhibition of folate absorption and metabolism. Concerning the first step, ethanol is responsible for repressing folate transporter gene expression through the methylation in CpG sites. Thus, alcohol consumers present a deficient folate absorption. Moreover, ethanol has been shown to produce an inhibitory effect on folate metabolism-related enzymes such as MTHFR and MTR (affecting *S*-adenosylmethionine pool and methylation activity in cells) and decreases *TYMS* mRNA levels. Otherwise, it increases *ALDH1L1* and *ALDH1L2* expression to alleviate ethanol-induced oxidative stress [41]. Nevertheless, despite its clear influence on breast cancer, the molecular mechanisms are not entirely deciphered. It has been proposed that it may be due to the mechanism previously described, increased levels of estrogen and its receptors derived from alcohol intake, and aromatase increased activity [36,42].

Alcohol’s influence on breast cancer and cell metabolism has been assessed in several studies. According to data reported by the study developed in the EPIC cohort, which included 360,000 women, alcohol consumption increases the risk of ER+ breast cancer [43]. Furthermore, the impact on DNA expression has been explored by the study conducted by Perrier et al. (2019) within the EPIC project. They concluded that alcohol intake is associated with methylation patterns at two CpG sites (cg03199996 and cg07382687) located in genomic regions associated with tumor suppressor activity (*GSDMD* and *HOXA5* genes) [44]. Additionally, the immune system intimately related gene, *Cd14*, presents expression changes in the presence of ethanol [45]. Concerning breast cancer, alcohol-induced genes *BRAF* and *ITPR1* presented a higher expression in ER+ breast cancer patients with a higher alcohol intake. Otherwise, alcohol-repressed genes *BAFT* and *ITPKA* had a higher expression in patients with the lowest alcohol intake [46]. According to alcohol metabolism-involved genes, three were up-regulated (*ITGA5*, *CBS*, and *SOD2*), and six were down-regulated (*XDH*, *XRCC1*, *MTHFR*, *CYP1B1*, *XPC*, and *GSTP1*) among women who died for breast cancer [47]. Nevertheless, alcohol has been associated with a higher TNBC cell proliferation, migration, and invasion through alcohol-induced reactive oxygen species and p38 and JNK phosphorylation, essential elements of the NK-κB signaling pathway [48].

### 2.2. Description of Genes Related to One-Carbon Metabolism

The 1CM features the confluence of three pathways: folate metabolism, methionine cycle, and trans-sulphuration. According to the current scientific literature, a total of 46 genes are related to 1CM. These genes are shown in Table 1. The information has been collected from the public repositories GeneCards^®^ 5.20 version (https://www.genecards.org/, accessed on 19 March 2024) [49] and Ensembl (https://www.ensembl.org/index.html, accessed on 19 March 2024) [50].

The 48 genes involved in 1CM are distributed across 20 chromosomes, although chromosome 5 contains the highest number of 1CM-related genes (*DHFR*, *MTRR*, *MAT2B*, *BHMT*, and *DMGDH*), followed by chromosomes 2, 11, and 21, with 4 genes in each one of them (*MTHD2*, *MAT2A*, *ATIC*, and *DNMT3A* in chromosome 2; *FOLR1*, *FOLH1*, *CBLIF*, and *TCN1* in chromosome 11; and *SLC19A1*, *CBS*, *FTCD*, and *GART* in chromosome 21) and chromosomes 1 and 19, which contain 3 genes each (*MTHFR*, *MTR*, and *CTH* in chromosome 1, and *DNMT1*, *PRMT1*, and *CD320* in chromosome 19).

### 2.3. Description of Single Nucleotide Polymorphisms Related to One-Carbon Metabolism

The search of genes related to 1CM in the GWAS Catalog provided a total of 706 SNPs associated with an outcome related to cancer at a *p*-value of 5 × 10^−8^. In addition, literature consultation of 1CM-related SNPs associated with breast cancer retrieved a total of 23 SNPs (Table 1). The study of minor allele frequencies (MAF) in the Caucasian population of the selected SNPs left 331 SNPs. Finally, 48 SNPs potentially related to both 1CM and cancer remained after restricting to linkage disequilibrium (LD) (R2 and/or D’ value < 0.7) between those SNPs located in the same chromosome (Table 2).

Most of the candidate genes remained with at least one potential SNP related to cancer. However, SNPs located in *CBS*, *FOLR1*, *AMT*, *GART*, *FPGS*, *MTFMT*, *SLC25A32*, *MAT2B*, *ATIC*, and *SHMT2* genes did not remain after the above-mentioned selection criteria. On the other hand, the *DNMT3A* (rs11890065, rs7581217, and rs752208), *CUBN* (rs1801222, rs61841503, and rs796667), and *SARDH* (rs2519125, rs2073817, and rs476835) were the ones with the highest number of selected SNPs. According to the type of variant, most of the SNPs selected were intronic variants (23 SNPs) followed by missense type variants (12 SNPs). Complete information about the risk allele, the *p*-value, and the outcome the SNPs are associated with is shown in Table S2.

### 2.4. Genes Functional and Enrichment Analyses

FUMA, REVIGO, ShinyGO, and R software package ClusterProfiler 4.12.0 in R Studio were the sources used for functional and enrichment analyses. These tools provided information about the 1CM-related gene expression patterns in different tissues and their function according to the molecular and cellular pathways in which they are involved. Additionally, information was obtained about the genes’ role in the underlying mechanisms of several diseases.

**Figure 2:**
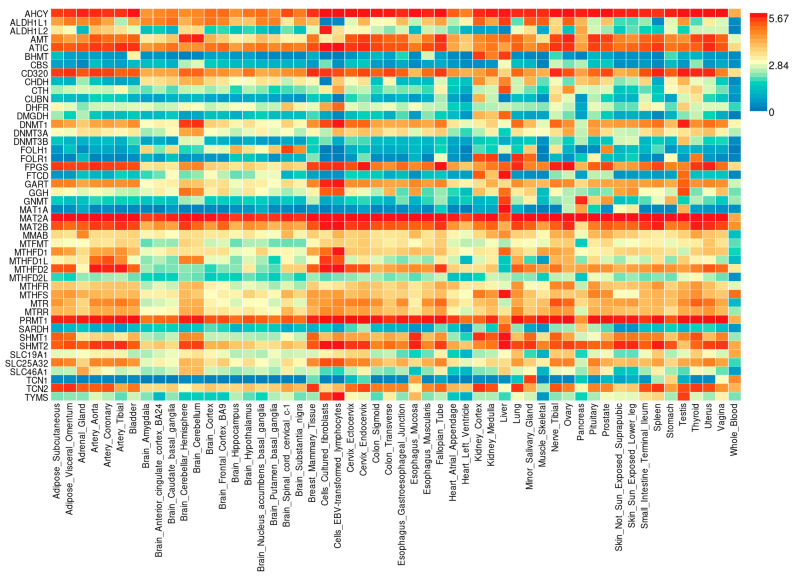
Heat-map of the one-carbon metabolism-related genes average expression pattern in different tissues and cells. The expression profiles are based on GTEx v8 RNA-seq data for 54 tissue and cell types. Scale bar represents gene expression measured in TPM (Transcripts Per Million). Cells in darker red mean higher expression of the gene compared to a darker blue color. This allows for comparison across tissue/cell labels and genes. Source: FUMA.

The FUMA tool supplied data on the expression pattern of the 1CM-related genes in different tissues or cells. Genetic expression profiles are illustrated in a heat-map shown in Figure 2. We observed a higher expression pattern along all tissues and cells for *AHCY*, *MAT2A*, *MAT2B*, *ATIC,* and *PRMT1* genes, while the genes *BHMT*, *CBS*, *FOLH1*, *FOLR1*, *TCN1*, *DNMT3B*, and *MAT1A* had a smaller tissular expression compared with the interrogated genes. Interestingly, 1CM-related genes with the higher expression pattern in the assessed tissues or cells participate in the methionine cycle (*AHCY*, *MAT2A,* and *MAT2B*), the biosynthesis of purines (*ATIC*), and the methylation of biological substrates different from DNA (*PRMT1*). Nevertheless, those genes with a reduced expression pattern are involved in the absorption of folate (*FOLH1* and *FOLR1*), the methionine cycle (*BHMT* and *MAT1A*), trans-sulphurization pathway (*CBS*), DNA methylation (*DNMT3B*), and cobalamin metabolism (*TCN1*).

Concerning the gene expression pattern in the tissues or cells included, cultured fibroblasts were the biological matrix with the biggest number of highly expressed genes (*AHCY*, *ATIC*, *CD320*, *DHFR*, *DNMT1*, *FPGS*, *GART*, *GGR*, *MAT2A*, *MAT2B*, *MATHFD1*, *MTHDF1L*, *MTHFD2*, *PRMT1*, *SHMT2*, *SLC25A32*, and *TYMS*) as well as testis (*AHCY*, *ALDH1L*, *AMT*, *ATIC*, *CD320*, *DNMT1*, *FPGS*, *FTCO*, *GART*, *GGH*, *MAT2A*, *MAT2B*, *MTR*, *PRMT1*, *SHMT1*, *TCN2*, and *TYMS*) both presenting 17 genes with higher expression than the rest of the 1CM-related genes.

Additionally, in vitro B lymphocytes transformed by the Epstein–Barr virus (EBV) showed a high expression patter of the genes *AHCY*, *ATIC*, *CD320*, *DNMT1*, *FPGS*, *GART*, *GGH*, *MAT2A*, *MAT2B*, *MATHFD1*, *MATHFD1L*, *MATHFD2*, *PRMT1*, *SHMT2*, *SLC25A32*, and *TYMS;* followed by the liver where *AHCY*, *ALDH1L1*, *BHMT*, *CTH*, *FPGS*, *FTCD*, *GGH*, *GNMT*, *MAT1A*, *MAT2A*, *MTHFD1*, *MTHFS*, *SHMT1*, *SHMT2*, and *SARDH* presented a higher expression. Otherwise, the tissue with the lowest expression profile of the 1CM-related genes was the whole blood, in which the genes *ALDH1L1*, *BHMT*, *CBS*, *CHDH*, *CTH*, *CUBN*, *DMGDH*, *DNMT3B*, *FOLH1*, *FOLR1*, *FTCD*, *MAT1A*, *MTHFD2L*, *SARDH*, and *SLC46A1* had a lower expression than the others. In addition, heart-related tissues present a lower expression of the 1CM-involved genes. Precisely, the heart’s left ventricle has a lower expression pattern for the genes *ALDH1L2*, *CBS*, *DMGDH*, *FTCD*, *GNMT*, *MAT1A*, *GGH*, *SARDH*, *MTHFD1L1*, *MTHFD1L2*, *SLC19A1*, *SLC46A1*, *TCN1,* and *TYMS*. Furthermore, the genes set with the lower expression patter in the heart atrial appendage tissue are *BHMT*, *CBS*, *CUBN*, *DMGDH*, *DNMT3B*, *FOLH1*, *FOLR1*, *FTCD*, *MAT1A*, and *TCN1*.

Focusing on the breast mammary tissue, *AHCY*, *AMT*, *ATIC*, *CD320*, *DNMT1*, *FPGS*, *GART*, *MAT2A*, *MAT2B*, *MTHFD1*, *MTHFD2*, *PRMT1*, *SHMT1*, *SHMT2*, *SLC25A32*, and *TCN2* were the 1CM-related genes most highly expressed. On the other hand, the genes *BHMT*, *CBS*, *FTCD*, *MAT1A*, and *SARDH* were the ones with the lowest expression pattern.

**Figure 3:**
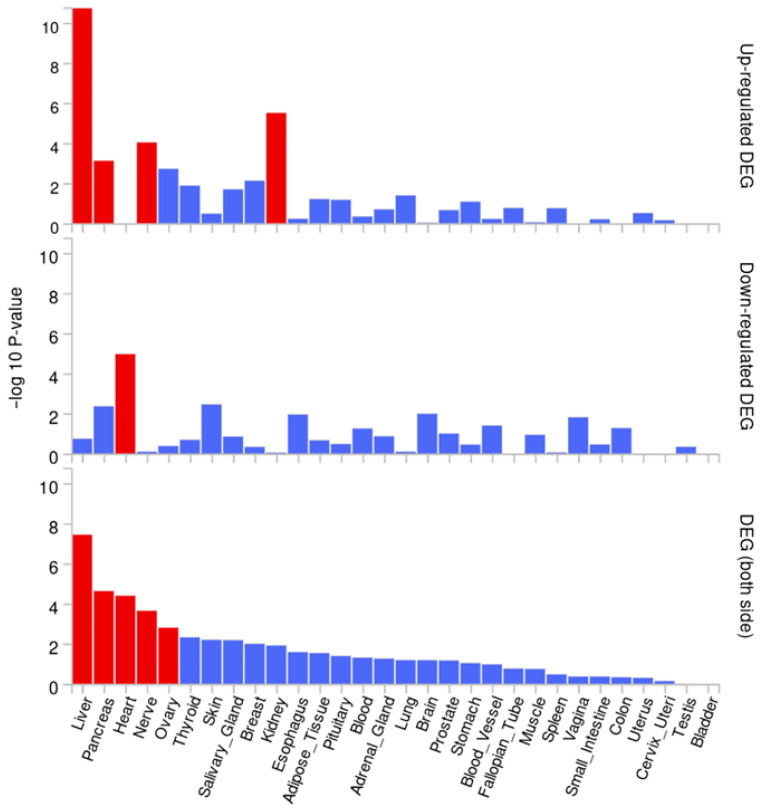
Tissue specificity expression of one-carbon metabolism-related genes in different tissues. The upper diagram shows the up-regulation of the 1CM-related genes in different tissues; meanwhile, the second diagram refers to the tissues where the genes involved in 1CM are down-regulated. Finally, the bottom diagram shows the differential expression of the genes (DEG) of the interrogated genes in all the tissues. Input genes were tested against each of the DEG sets using a hypergeometric test. The background genes are those that have an average expression value > 1 in at least one of the tissue labels and exist in the selected background genes (all). Tissues with significant enrichment at Bonferroni corrected *p*-value ≤ 0.05 are colored in red. Bonferroni correction is performed for each of the up-regulated, down-regulated, and both-sided DEG sets separately. Source: FUMA.

Additionally, the joint expression of the 1CM-related genes differs between tissues, as it is shown in Figure 3. Those tissues where the 1CM-involved genes are up-regulated with a significant enrichment *p*-value after Bonferroni correction are the liver, the kidney, the nerves, and the pancreas, followed by the ovary and breast, although the enrichment is not significant. Otherwise, the down-regulated differential expression of the genes (DEG) occurs significantly in the heart, followed by the pancreas, the skin, the esophagus, and the brain, albeit the *p*-value of these last tissues was not significant. Concerning both sides, the liver is the tissue with the highest enrichment *p*-value for DEG of those genes involved in 1CM compared to other tissues. Furthermore, the pancreas, heart, nerve, and ovary also present a significant enrichment *p*-value of the 1CM-related gene DEG compared to the rest of the interrogated tissues. Conversely, tissues engaged in the digestive, excretory, and reproductive apparatus were the ones with the lowest DEG value of the studied genes (small intestine, colon, uterus, cervix uteri, testis, and bladder).

Focusing on breast tissue, it presents a higher proportion of up-regulated genes. Indeed, the breast is the tissue with one of the lowest DEG *p*-values within the down-regulated analysis. Considering both sides, breast tissue presents a higher expression of the 1CM-related genes than most of the retrieved tissues.

More detailed information about the number of background and overlapped genes and the adjusted and non-adjusted *p*-value for each tissue can be consulted in Table S3.

The enrichment and functional analyses were performed on the Kyoto Encyclopedia of Genes and Genomes (KEGG), WikiPathways, MsigDB, and GeneOntology (GO) via the bioinformatic tools ShinyGO, FUMA, REVIGO, and the software R package ClusterProfiler 4.12.0 in R Studio. The genes listed in Table 1 compared to the reference genome (57,241 genes) were used in these analyses. Some insights about the main mechanisms and signaling pathways in which the 1CM-related genes are involved were obtained, as well as the main genetic and biochemical alterations in which they participate.

**Figure 4:**
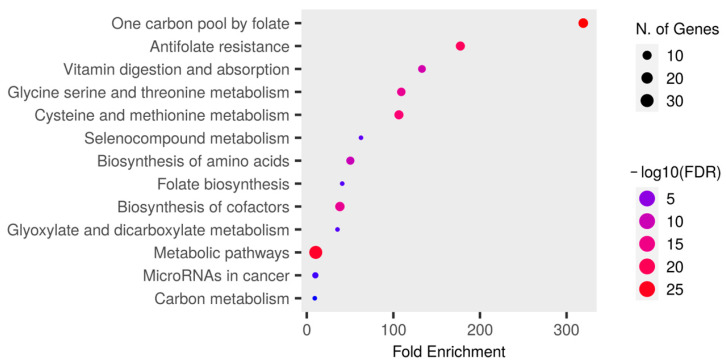
Functional and enrichment analysis for the one-carbon metabolism-related genes in the cellular and molecular mechanisms from GeneOncology. In the diagram above is represented the association of the one-carbon-related genes and the different mechanisms in which they are involved after FDR correction. Dot size refers to the number of genes involved in the pathway. FDR value is defined through a color scale. Thus, the closer to red, the bigger the association is, and the reverse for blue. Fold enrichment is defined as the percentage of the selected genes involved in one pathway divided by the percentage of the reference genes. It assesses the enrichment magnitude, so a higher value means a stronger enrichment. Source: ShinyGO.

Cells phenomena with a significant *p*-value (<0.05, after false discovery rate (FDR) correction) in the enrichment analysis are shown in Figure 4. The pathways in which these genes are involved were as follows: one-carbon pool by folate, antifolate resistance, vitamin digestion, and absorption; glycine, serine, and threonine metabolism; cysteine and methionine metabolism; selecompounds metabolism; biosynthesis of amino acids; folate biosynthesis; biosynthesis of cofactors; glyoxylate and dicarboxylate metabolism; metabolic pathways; microRNAs (miRNAs) in cancer; and carbon metabolism. Regarding the results of the enrichment analysis, the interrogated genes were mainly related to the one-carbon pool by folate, showing a high association and enrichment value. Furthermore, the analysis performed revealed an association between the 1CM-related genes and the nitrogen metabolism via several amino acids’ metabolism and biosynthesis (serine, glycine, threonine, cysteine, and methionine) with a similar number of genes involved and −log10 FDR value. Additionally, it was observed that, despite a low proportion of participating genes, the term “metabolic pathways” had a high −log10 FDR value. This term is defined as reactions and mechanisms that transform molecules, including macro-molecular processes such as DNA repair, replication, and methylation, which are essential in the carcinogenic process.

**Figure 5:**
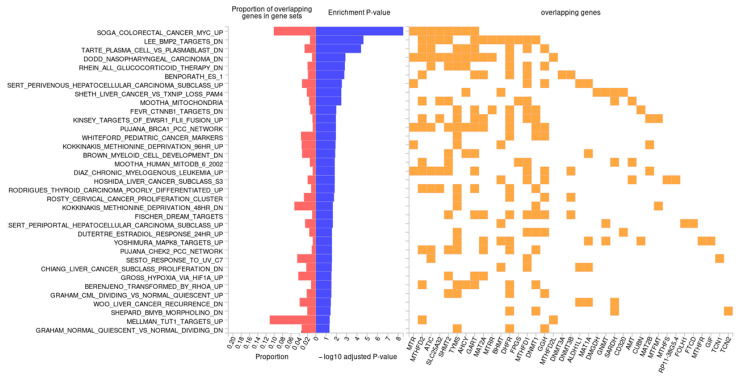
In silico enrichment analysis in the one-carbon metabolism-related genes on the main genetic and biochemical alterations (MsigDB). Figures were created according to the comparison between the interrogated genes and the reference genome through KEGG and MsigDB. The overlapping genes related to one-carbon metabolism (in yellow), the FDR *p*-value after the enrichment analysis (in blue), and the proportion of overlapping genes compared to the reference genome in each associated pathway (in red) are shown. Source: FUMA.

Likewise, the 1CM-related genes may be involved in biochemical and genetic alterations that are the basis of various pathologies. According to the analysis carried out using the FUMA and REVIGO tools, the genes are related to a wide variety of these alterations. Regarding genes involved in the different pathways, a large proportion of them were involved in signaling pathways linked to carcinogenesis, mediated by *MAPK*, *RAS*, *HOX11*, *TP53,* and *MYC* [51,52,53]. There was also a high proportion of genes involved in processes related to different types of cancer. Furthermore, other cellular processes related to the interrogated genes are involved in pathways associated with cell adaptation to stress and hypoxia through the hypoxia-inducible factor 1-α, which is associated with breast cancer response to chemotherapy [54,55]. On the other hand, the results of the enrichment analysis also linked the genes of interest to mechanisms involved in other pathologies with glucocorticoid therapy (Figure 5). According to the FDR value obtained, the main mechanisms involving the interrogated genes were those responsible for nasopharyngeal carcinoma and colorectal cancer mediated by *MYC* overexpression (Figure 5).

Finally, Figure 5 shows information about the overlap of certain genes in the different cellular mechanisms. Processes with the biggest number of overlapped genes were related to uterus adaptation during pregnancy (*MTHFD2*, *ATIC*, *SLC25A32*, *SHMT2*, *GART*, *MAT2A*, *MTRR*, *BHMT*, *DHFR*, *FPGS*, and *MTHFD1*) [56], and nasopharyngeal carcinoma (*MTR*, *MTHFD2*, *ATIC*, *SLC25A32*, *SHMT2*, *TYMS*, *AHCY*, *GART*, *MAT2A*, *MTRR*, *DHFR*, and *MTHFD2L*) [52]. Additionally, the third pathway with the highest number of overlapping genes was found regarding the *BCRA1*-Pearson correlation coefficient (PCC) network [57], this model being potentially associated with breast cancer and the *BRCA1* mutations effect. The overlapping genes in this network were *MTR*, *MTHFD2*, *ATIC*, *SHMT2*, *TYMS*, *AHCY*, *GART*, *MAT2A*, *DHFR*, *GGH*, and *DNMT1*. Furthermore, alterations related to carcinogenesis in the colon and rectum had a high proportion of overlapping genes in the gene-sets.

More detailed information about the number of background and overlapped genes for each genetic and biochemical alteration in the adjusted and non-adjusted *p*-value from the enrichment analysis can be consulted in Table S4.

On the other hand, enrichment analyses, according to the overall and disease-free survival of breast cancer, have been performed using the online tools GEPIA and UALCAN [58,59]. These online free-use platforms provide information about the expression of those genes involved in survival for a precise type of cancer according to the data from the TCGA Study (The Cancer Genome Atlas Program, National Cancer Institute, NCI) [60].

**Figure 6:**
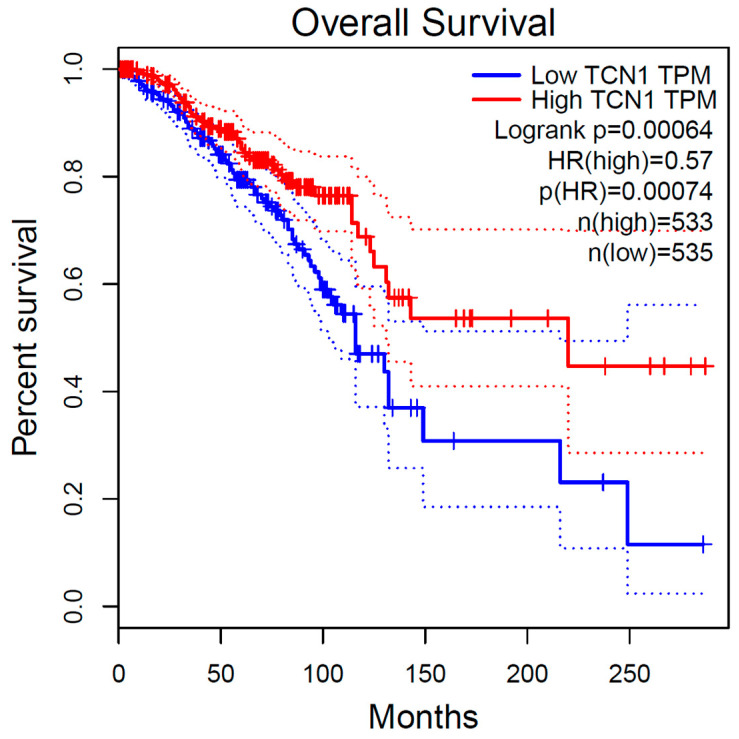
Kaplan–Meier plot of overall survival curves from in silico enrichment analysis according to *TCN1* expression in breast cancer patients from TCGA Study. Source: GEPIA.

**Figure 7:**
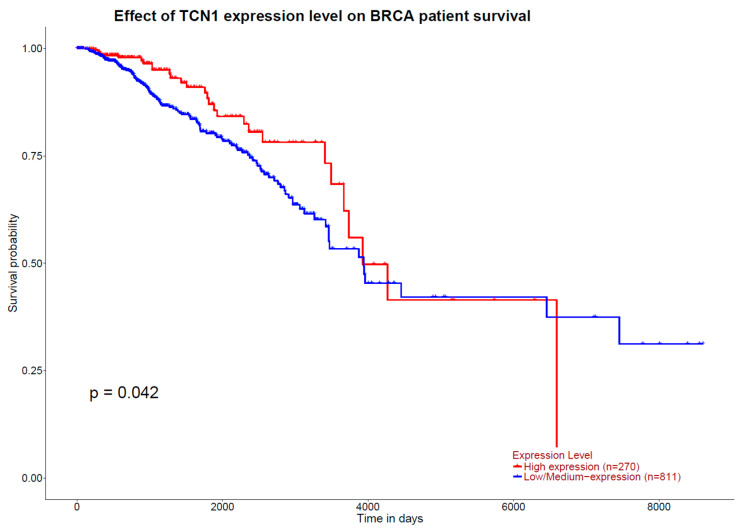
Kaplan–Meier plot of overall survival curves from in silico enrichment analysis according to *TCN1* expression in patients diagnosed with invasive breast carcinoma. Source: UALCAN.

**Figure 8:**
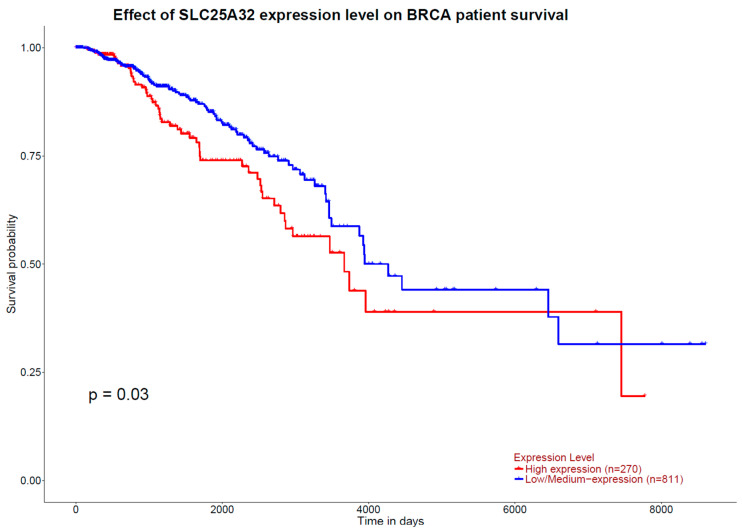
Kaplan–Meier plot of overall survival curves from in silico enrichment analysis according to *SLC25A32* expression level in patients diagnosed with invasive breast carcinoma. Source: UALCAN.

**Figure 9:**
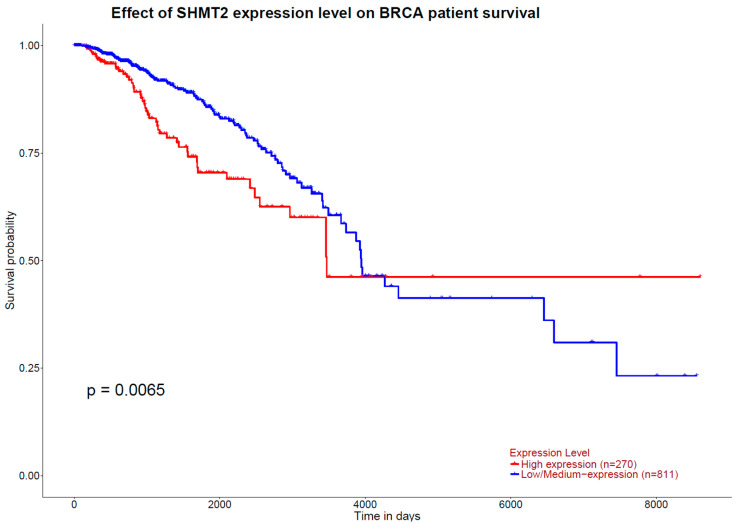
Kaplan–Meier plot of overall survival curves from in silico enrichment analysis according to *SHMT2* expression level in patients diagnosed with invasive breast carcinoma. Source: UALCAN.

GEPIA results indicate that higher expression of the 1CM-participating gene *TCN1*, measured in TPM, is associated with a higher overall survival rate in patients with breast cancer (*p*_logRank_ = 0.0007) (Figure 6). Inversely, concerning breast cancer free-disease survival, none of the 1CM genes showed any association. These results are in accordance with the ones obtained from the tool UALCAN, where a higher expression of *TCN1* is observed in those patients diagnosed with breast invasive carcinoma and with a higher overall survival (*p* = 0.042) (Figure 7). Otherwise, UALCAN results indicate that expression of *SLC25A32* and *SHMT2* correlates with overall survival in breast invasive carcinoma. Indeed, a higher expression of *SLC25A32* is associated with a poorer overall survival rate of the patients compared to those with a lower expression (*p* = 0.03) (Figure 8). Furthermore, results for *SHMT2* indicate that a lower expression of the gene is associated with a low overall survival rate (*p* = 0.0065) (Figure 9).

## 3. Discussion

In this study, the association between nutrients and genes involved in 1CM and cancer, specifically breast cancer, was investigated. Several computational tools and biological databases have been used to identify the molecular, metabolic, and cellular mechanisms linking the genes of interest to cancer.

The results of the literature search highlight the role of vitamins B2, B6, B9, and B12, as well as alcohol consumption, in the development of breast cancer. Current scientific evidence indicates that higher intakes of B2, B6, and folate decrease the risk of developing breast cancer. On the other hand, there is controversy about the effect of plasma levels of these nutrients. As for alcohol, it has been observed that any consumption favors the onset of the disease. The results of the in silico functional and enrichment analysis show that the genes of interest are up-regulated in the breast tissue compared to other tissues and cells. Precisely, genes *AHCY*, *CD320*, *FPGS*, *MTHFD2*, *PRMT1*, and *TCN2* present an outstanding expression compared to the rest of the interrogated genes. Additionally, they are involved in a wide variety of cellular processes and metabolic pathways, 1CM being the main one, as well as the metabolism of several amino acids and the digestion and absorption of proteins, antifolate resistance, and miRNAs in cancer. Current scientific evidence indicates that miRNAs play an important role in cancer, being involved in the regulation of different cancer mechanisms such as apoptosis, TNFα signaling, hypoxia, or inflammatory response. Furthermore, a wide range of miRNAs has been related to breast cancer, either acting as tumor suppressors (e.g., *MIR100*, *MIR1-1,* or *MIR114*) or oncogenes (e.g., *MIR115*, *MIR17*, or *MIR224*) [61]. Furthermore, survival enrichment analyses between the 1CM-related genes and survival in breast cancer indicate that a higher expression of *TCN1* correlates with a higher overall survival. Conversely, genes *SLC25A32* and *SHMT2* higher expression is associated with poorer overall survival. The above-mentioned mechanisms are closely related to cell proliferation and carcinogenesis. In addition, the 1CM genes are involved in various biochemical and genetic alterations that cause several pathologies, including cancer, mainly colorectal, nasopharyngeal, and breast cancer. Indeed, expression of 1CM-related genes as *ALDH1L2* has been associated with the response to chemotherapy with 5-FU (5-fluorouracil, inhibitor of folate metabolism) in colorectal cancer patients [62]. Additionally, it has been reported that participating nutrients in 1CM (e.g., methionine and betaine) may influence colorectal cancer risk [63]. On the other hand, the role of the 1CM-related genes and nutrients has not been already explored, albeit the nasopharyngeal carcinoma network elucidates that the disease is closely related to mechanisms where 1CM plays a pivotal role, such as DNA repair [52]. Concerning breast cancer, the 1CM-involved genes are highly overlapped in the alterations derived from the breast cancer network, which uses *BRCA1*, *BRCA2*, *CHEK2,* and *ATM* as reference genes for the disease. The network connects a wide range of genes involved in breast cancer after PCC, where we can find 11 genes related to 1CM. Interestingly, the breast cancer reference gene *ATM* is also one of the 1CM-involved genes [57]. Thus, molecular variants in the genes studied may influence the development and survival of cancer and, specifically, breast cancer. Wu et al. (2016) measured genome stability and cell viability in vitro in lymphocytes from women with breast cancer and healthy controls as a function of the expression of the 1CM-related genes (*SHMT*, *MTR,* and *MTRR*) and vitamin B6. They observed a positive correlation between vitamin B6 and genome stability, with 48 nmol/L being the optimal vitamin B6 concentration. In addition, they indicated that SNPs located in the genes studied are involved in the stress to which the cell is subjected [64].

This is the first study on functional and enrichment analyses between genes involved in 1CM and cancer, focusing on breast cancer. Currently, few in silico analyses on the main potential possibly involved in breast cancer have been published. These functional analyses agree on the association of a few genes with breast cancer survival and prognosis, such as *DLGAP5*, *NCAPG*, and *RRM2*, which are not involved in 1CM [65,66].

Some previous studies have evaluated the role of B2, B6, B12, folate, folic acid, and alcohol consumption, as well as the influence of SNPs located in these genes on breast cancer.

The study conducted by Maruti et al. (2009) evaluated the role of the *MTHFR* rs1081133 polymorphism, as well as folate, B2, B6, B12, and alcohol intake in 318 breast cancer cases and 647 controls of European Caucasian origin, matched for age and race. The results of the study indicated that women carrying the TT genotype of these genes have a higher risk of developing breast cancer after menopause (OR = 1.62; 95%CI = 1.05–2.48; *p* < 0.05). Furthermore, lower folate intake combined with *MTHFR* rs1081133 contributed to an even higher risk [67], thus supporting an interaction between folate intake and this SNP of the *MTHFR* gene. On the other hand, the study by Ma et al. (2009), where the role of the SNPs *MTHFR* rs1081133 (G > A) and rs1081131, and *MTR* rs1805087, as well as the dietary intake of folate, B6 and B12 was explored among 458 women with breast cancer and 458 women without the disease from Brazil (mixed origin), showed that the GG genotype of the *MTR* rs1805087 increases the risk of overall breast cancer (OR = 1.99, 95%CI = 1.01–3.92; *p* = 0.01), while no association was observed for the *MTHFR* SNP rs1081133. Regarding the intake of the B-complex vitamins involved in 1CM, the results of this study do not agree with those of previous studies since women with a higher intake of folate had a higher risk of developing breast cancer, mostly among premenopausal women (OR = 2.17, 95%CI = 1.23–3.82, *p* = 0.01) [68].

Taking into account the development of breast cancer as a function of hormone receptors, in the study carried out by Wang et al. (2022) in 439 women with breast cancer (and 439 controls) of Asian origin, the influence of the *MTR* rs1805087, *MTHFR* rs1801133, *ALDH1L1* rs2002287 (G > A), *DNMT1* rs2228611, and *DNMT3B* rs2424908 (C > T) variants on breast cancer was examined. Results showed that the A allele of *DNMT1* rs2228611 decreased the risk of overall breast cancer (OR = 0.74; 95%CI = 0.56–0.97; *p* = 0.03; GA + AA vs. GG). This SNP was also associated with a lower risk of ER+, PR+, and HER2 breast cancer. Similarly, the stratified analysis by hormone receptors indicated that carriers of the C allele of *ALDH1L1* rs2002287 present an increased risk of developing breast cancer PR+ (OR = 1.54; 95%IC = 1.04–2.26; *p* = 0.03) [69]. Interestingly, this gene is also associated with alcohol metabolism, an established risk factor for breast cancer.

Similarly, studies have considered the adherence to a dietary pattern and the interaction with the 1CM-related SNPs to assess their influence on breast cancer. Cao et al. (2021) conducted this study on 818 breast cancer cases and 935 controls of Asian origin. The results showed no association between SNPs, studied individually, and breast cancer. However, taking into account the joint effect of all SNPs using a polygenic risk score or PRS, an increased risk of overall breast cancer was observed (OR = 2.09, 95%CI = 1.54–2.85, *p* < 0.001). On the other hand, greater adherence to the Mediterranean diet was associated with a lower risk of postmenopausal breast cancer (OR = 0.54, 95% CI = 0.38–0.78, *p* = 0.001). Considering the interaction between the Mediterranean diet and PRS, an increased risk of overall and postmenopausal breast cancer was observed [70]. This finding also highlights a potential interaction between dietary factors and 1CM-related SNPs.

On the other side, few studies have assessed the role of the 1CM-involved genes in breast cancer survival. The study developed by Xu et al. (2008) enrolled 1479 breast cancer cases where they assessed the role of B-complex vitamins dietary intake and 1CM SNPs on survival. Results showed that the altered allele of *MTHFR* rs1801133 (T) carriers have reduced all-cause mortality (HR = 0.69, 95%CI = 0.49–0.98) and breast cancer-specific mortality (HR = 0.58, 95%IC = 0.38–0.89) [71]. The association of 1CM with cancer survival is closely related to chemotherapy. Indeed, Zhang et al. (2024) assessed the role of 1CM in breast cancer response to treatment using the GSE20685 from the Gene Expression Omnibus (GEO) dataset. The risk score model was constructed and included the 1CM genes *MAT2B*, *DNMT3B*, *CHDH*, *AHCY*, and *SHMT2*. Kaplan–Meier analysis revealed that *SHMT2* and *DNMT3B* can be labeled as risk factors for survival and were up-regulated in high-risk patients. By contrast, the analysis identified *MAT2B*, *AHCY*, and *CHDH* as protective factors, being down-regulated in high-risk patients [72].

The current study has limitations inherent to in silico studies, such as the lack of association of the genes studied with certain outcomes due to the limited information available in biological or genetic databases and repositories. In addition, we have not considered gene–environment interaction as a potential driver of breast cancer, which could have affected the final result. Furthermore, with respect to the observational studies, limitations that may have affected the conclusions drawn can be related to sample size, dietary assessment methods, participants’ ethnicity, and study design. Regarding the strengths of the study, we have used several sources and web tools to explore the relationship between genes and breast cancer, as well as their molecular activity and the metabolic pathways in which they participate. In addition, a selection has been made of those SNPs located in genes involved in 1CM and potentially associated with cancer, considering the previous scientific literature, the MAF in the Caucasian population, and the LD.

## 4. Materials and Methods

### 4.1. Search Strategy to Review the Relationship between Folate, B2, B6, B12, and Alcohol with Breast Cancer

A bibliographic search of the scientific literature has been performed to review the relationship between the nutrients and diet components related to 1CM and breast cancer. We conducted searches in Medline (PubMed) (https://pubmed.ncbi.nlm.nih.gov/, accessed on 20 March 2024) and Scopus (https://www.scopus.com/search/form.uri?display=basic#basic, accessed on 22 March 2024). The search strategy was as follows:

Breast[title] AND (carcinoma* OR cancer* OR malign* OR neoplasm* OR tumor* OR tumour*)
AND
(“vitamin B2” OR B2 OR riboflavin)
AND
(“vitamin B6” OR B6 OR pyridoxine OR pyridoxamine OR pyridoxal)
AND
(“vitamin B9” OR B9 OR folate OR “folic acid”)
AND
(“vitamin B12” OR B12 OR cobalamin)
AND
alcohol

### 4.2. Annotation of the Genes Related to One-Carbon Metabolism and Single Nucleotide Polymorphisms Selection

To select the genes related to 1CM, we performed a search in the main scientific databases Medline (PubMed) (https://pubmed.ncbi.nlm.nih.gov/, accessed on 9 March 2024), Scopus (https://www.scopus.com/search/form.uri?display=basic#basic, accessed on 9 March 2024) and Gene (National Health Institutes, NIH, accessed on 9 March 2024) (https://www.ncbi.nlm.nih.gov/gene, accessed on 9 March 2024) following the search strategy shown below:

“One-carbon metabolism”[title] AND (gene* OR genome*)

Information about the selected genes shown in Table 1 was collected by consultation of the public repositories GeneCards^®^ 5.20 version (https://www.genecards.org/, accessed on 10 March 2024) and Ensembl (https://useast.ensembl.org/index.html, accessed on 10 March 2024). GeneCards^®^ is an integrative database that provides comprehensive information about human genes and their related diseases, variants, proteins, cells, and biological pathways. The knowledgebase integrates information from more than 150 sources (e.g., Alliance of Genome Resources, Atlas, ClinVar, Disease Ontology, GO, GeneBank, GENATLAS, OMIM, etc.) [49]. Ensembl belongs to the Ensembl project, started in 1999, whose objective was to annotate the genome and integrate the information with other available biological data. The website offers information about the genome of vertebrates and model species [50].

In parallel, the SNPs related to 1CM and breast cancer were selected considering the scientific evidence about the SNPs and their relationship with any cancer-related outcome. For this purpose, we used the database GWAS Catalog (https://www.ebi.ac.uk/gwas/ home, accessed on 11 March 2024), which offers a curated collection from all high-quality published GWAS studies [73]. Additionally, this source allows the cross-reference of SNPs and other genetic variants with a wide range of phenotypes. We submitted the selected genes (Table 1) to identify those SNPs associated with any global cancer-related outcome and whose *p*-value was lower than 5 × 10^−8^. Furthermore, we performed a bibliographic search of the SNPs located in the 1CM genes that have been specifically associated with breast cancer, also using the databases PubMed and Scopus. The search strategy was as follows:

Breast[title] AND (carcinoma* OR cancer* OR malign* OR neoplasm* OR tumor* OR tumour*)
AND
“one-carbon metabolism”
AND
(“single nucleotide” OR gene* OR genom* AND (polymorphism* OR variant* OR variation*))

Subsequently, we studied the MAF of the selected SNPs in the Caucasian population, according to the published data in Ensembl (https://www.ensembl.org/index.html, accessed on 14 March 2024) [50]. Those SNPs with a MAF lower than 1% in the reference population were considered rare mutations and removed from the selection. Finally, LD was studied in the remaining SNPs with the online tool LDlink (NIH, https://ldlink.nih.gov/?tab=ldmatrix, accessed on 15 March 2024) [74] using the genome version GRCh38.p14 as a reference to study all closely related variants within the same chromosome. LDlink repots information about the pairwise LD statistics for those SNPs located in the same chromosome with an interactive matrix. The SNPs with an R2 and/or D’ value higher than 0.7 were considered in LD and removed from the selection.

### 4.3. Biological Database Studies

We performed functional and enrichment analyses of the genes selected in the previous steps using online computational biology tools and R software packages (Clusterprofiler 4.12.0) [75,76], which are described below.

#### 4.3.1. In Silico Functional Analyses and Enrichment Analyses

The bioinformatic tools used were FUMA (Functional Mapping and Annotation of Genome-Wide Association Studies), REVIGO, ShinyGO, GEPIA, and UALCAN. These databases contain genetic information from several sources such as Ensembl, Gene Ontology (GO), and the Kyoto Encyclopedia of Genes and Genomes (KEGG).

FUMA is an online platform to prioritize, visualize, and interpret GWAS results [77]. This tool provides useful functional biological information. Additionally, it performs functional and enrichment analyses based on genes, molecular pathways, and specific tissue information. The sources of information for FUMA are GO, WikiPathways, and MsigDB. Using the module GENE2FUNC (https://fuma.ctglab.nl/gene2func, accessed on 20 March 2024), we obtained information about the gene expression pattern in several tissues, as well as the main molecular and cellular mechanisms in which they are involved. Additionally, this tool allows us to know those pathologies with which the selected genes are associated. In the in silico functional analyses, we compared the genes listed in Table 1 with the background gene-set. Ensembl v102 and GTEx v8 were used to obtain the genetic and tissular information. Gene expression heat-map (Figure 2) gives an averaged expression per tissue/cell label per gene following winsorization at 50 and log2 transformation with a pseudocount of 1. Furthermore, tissue specificity observed in Figure 3 is tested using the differentially expressed genes for each tissue label of each expression dataset. DEG sets were pre-calculated by performing a two-sided *t*-test for any one of the label tissues against all others. For this, expression values were normalized (zero-mean) following a log2 transformation of expression value (TPM). Genes with *p*-value ≤ 0.05 after Bonferroni correction and absolute log fold change ≥ 0.58 were defined as differentially expressed genes in a given tissue label compared to others. On top of DEG, up-regulated DEG and down-regulated DEG were also pre-calculated by taking the sing of t-statistics into account. Otherwise, the association in the enrichment analyses was calculated through Fisher’s exact test, and we considered significant *p*-values as those equal to or lower than 0.05 after the Benjamini–Hochberg (FDR) correction.

ShinyGO (http://bioinformatics.sdstate.edu/go/, accessed on 22 March 2024) is a bioinformatic tool developed by the South Dakota University powered by R packages such as Bioconductor 3.10 [78] in order to offer functional enrichment analyses from a gene list providing graphics and images of the related pathways, genes characteristics, and their interaction with proteins. ShinyGO gathers information from public repositories such as GO, Ensembl, and KEEG. Characteristics of the selected genes involved in 1CM were compared with the reference gene-set (57,241 genes) through the χ^2^ test and the Student’s *t*-test. FDR association *p*-values equal to or lower than 0.05 were considered significant. This tool offers information about the molecular mechanisms, chromosomic location, genetic interaction, the guanine–cytosine (GC) content, the 3′ UTR and 5′ UTR length, and the coding sequence of the listed genes. To perform the functional analysis, the genes Ensembl ID is required, as shown in Table 1.

REVIGO (http://revigo.irb.hr/, accessed on 26 March 2024) is a web platform that requires GO terms obtained from previous enrichment analyses [79]. The main function of this tool is the visualization of the results and their interpretation. REVIGO uses information from the databases GO and UniPro-to-Go mapping from the EBI GOA project. Additionally, this tool uses a clustering algorithm based on the semantic similitude of the terms, helping the results interpretation. All the GO terms were obtained from previous enrichment analyses on the listed genes in Table 1, using the R software package Clusterprofiler 4.12.0 in R Studio 2024.04.2+764 version [75,76].

The Gene Expression Profiling Interactive Analysis (GEPIA) (http://gepia.cancer-pku.cn/index.html, accessed on 10 July 2024) [58] is an interactive web server for analyzing the RNA sequencing expression data of 9736 tumors and 8587 normal samples from the TCGA [60] and GTEx project [80], using a standard processing pipeline. GEPIA provides customizable functions such as tumor/normal survival studies, which are performed through Cox regression analysis and plotted using Kaplan–Meier graphics.

The University of Alabama at Birmingham Cancer data analysis Portal (UALCAN) (https://ualcan.path.uab.edu/index.html, accessed on 11 July 2024) [59] is a user-friendly and interactive web tool that allows researchers to analyze cancer OMICS data. UALCAN is designed to provide easy access to publicly available data from the TCGA, MET500, CPTAC, and CBTTC, allowing users to identify biomarkers or perform in silico validation of potential genes of interest. Additionally, UALCAN allows the analysis of expression profiles and patient survival information for protein-coding, miRNA-coding, and lincRNA-coding genes and allows the evaluation of epigenetic regulation of gene expression by promoter methylation. Survival information is obtained through Cox regression and Kaplan–Meier plots.

#### 4.3.2. Workflow of the Analyses

**Figure 10:**
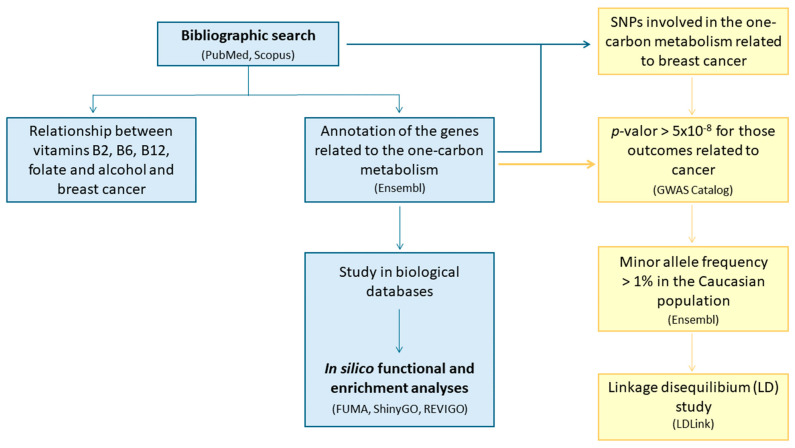
Workflow of the bibliographic search and data analysis.

We started the analysis with a bibliographic search on the association between 1CM and breast cancer to annotate the genes involved in this pathway and consider the dietary components related to 1CM. To select those SNPs potentially associated with cancer, we looked for variants located in the genes involved in 1CM from the GWAS Catalog (*p*-value threshold 5 × 10^−8^) and other databases (PubMed and Scopus). Subsequently, we consulted Ensembl to study the MAF of the SNPs in the Caucasian population. Additionally, the LD of the selected molecular variants was examined. In addition, we carried out an in silico functional and enrichment analysis study using biological databases such as FUMA, REVIGO, and ShinyGO (Figure 10).

## 5. Conclusions

In the present study, genes and SNPs related to 1CM that may have a potential role in cancer development, focusing on breast cancer, have been analyzed using information available in public biological databases via in silico enrichment and functional analysis. Likewise, bibliographic research on the current evidence about the role of B group vitamins involved in 1CM and alcohol on breast cancer has been conducted. Genes related to 1CM have been poorly explored regarding their role in breast cancer despite their potential involvement in this disease, as evidenced in biological databases. This study unveils that 1CM-related genes participate in a wide range of cellular mechanisms closely related to carcinogenesis, such as amino acid metabolism and cell proliferation, with a notable impact on breast cancer. According to the dietary compounds, vitamins B2, B6, B9, and B12 might be involved in breast cancer as well. Further molecular and nutritional studies are needed to elucidate the underlying linking mechanisms and to explore their potential interplay. Additionally, studies should evaluate the role of the SNPs selected in this study, as well as their interaction with nutrients involved in 1CM or foods rich in these nutrients in the etiology and survival of breast cancer.

## Figures and Tables

**Table 1:** Genes related to one-carbon metabolism: chromosomic location, gene size from the GRCh38.p14 genome version, the Ensembl ID, and function.

Gene	Chromosomic Location (Gene Size)	Ensembl ID	Function
*FOLR1*	11q13.4 (6766 bp)	ENSG00000110195	The coding protein is representative of the folate receptors family. Folate and folic acid bind to these receptors to enter the cell
*GGH*	8q12.3 (24,527 bp)	ENSG00000137563	The gene codifies for the γ-glutamyl hydrolase. It is responsible for hydrolyzing the polyglutamates from the diet where folate is bound
*FOLH1*	11p11.12 (63,547 bp)	ENSG00000086205	The folate hydrolase 1 is a transmembrane glycoprotein with glutamate carboxypeptidase activity for dietary folate
*SLC19A1*	21q22.3 (79,794 bp)	ENSG00000173638	The gene encodes a membrane protein that acts as a transporter of folate. The protein is involved in the regulation of folate intracellular concentration
*SLC25A32*	8q22.3 (16,470 bp)	ENSG00000164933	This gene leads to a member of the mitochondrial carrier family transport proteins. The encoded protein transports folate across the inner mitochondrial membrane
*SLC46A1*	17q11.2 (11,570 bp)	ENSG00000076351	This gene encodes a transmembrane proton-coupled folate transporter protein that facilitates the movement of folate and antifolate substrates across the cellular membrane
*DHFR*	5q14.1 (28,758 bp)	ENSG00000228716	The enzyme codified is the dihydrofolate reductase, which is involved in THF synthesis. Its deficiency has been related to megaloblastic anemia
*TYMS*	18p11.32 (15,926 bp)	ENSG00000176890	This gene codifies for the enzyme thymidylate synthase, the one in charge of maintaining the dTMP pool needed for DNA replication and repair
*MTHFD1*	14q23.3 (75,427 bp)	ENSG00000120254	The methyl-tetrahydrofolate dehydrogenase 1 presents three different enzymatic activities participating in the methionine, thymidylate, and purine synthesis
*MTHFD1L*	6q25.1 (236,209 bp)	ENSG00000120254	The resulting protein is involved in the THF biosynthesis in the mitochondrion. Several isoforms have been identified for this gene
*MTHFD2*	2p13.1 (31,394 bp)	ENSG00000065911	This gene codifies an enzyme with mitochondrial activity, which is involved in methionine and thymidylate synthesis. The enzyme is characterized for requiring Mg^2+^ and inorganic phosphate
*MTHFD2L*	4q13.3 (188,926 bp)	ENSG00000163738	This gene encodes a trifunctional enzyme that participates in the THF interconversion. It is predicted to be located in the mitochondrial matrix
*MTHFS*	15q25.1 (63,795 bp)	ENSG00000136371	The enzyme encoded by this gene catalyzes the conversion of 5-formyl-THF to 5,10-methenyl-THF. Two isoforms have been found for this gene
*SHMT1*	17p11.2 (35,704 bp)	ENSG00000176974	The gene codifies for the serine hydroxymethyltransferase 1, which participates in the methionine, thymidylate, and purines synthesis in cytosol
*SHMT2*	12q13.3 (5363 bp)	ENSG00000182199	This gene encodes for the serine hydroxymethyltransferase 2 in the mitochondrion. Enzyme activity has been suggested to be the primary source of intracellular glycine
*MTHFR*	1p36.22 (20,733 bp)	ENSG00000177000	This is a paralogue gene of *MTR*. It codifies for the methyl-tetrahydrofolate reductase that catalyzes the conversion of 5,10-mTHF of 5-mTHF, which is a co-substrate of homocysteine re-methylation to methionine
*MTR*	1q43 (126,019 bp)	ENSG00000116984	The gene codifies for the enzyme methionine synthase, which depends on cobalamin. It is in charge of catalyzing the final step of methionine biosynthesis
*MTRR*	5p15.31 (55,167 bp)	ENSG00000124275	The gene codifies for the 5-mTHF-homocysteine methyltransferase reductase, which is involved in methionine synthesis, regenerating the enzyme methionine synthase to a functional state
*MAT1A*	10q22.3 (17,839 bp)	ENSG00000151224	This gene leads to the enzyme methionine adenosyl-transferase, which is in charge of transferring adenosyl groups for methyl group generation
*MAT2A*	2p11.2 (6114 bp)	ENSG00000168906	The protein encoded by this gene catalyzes the production of *S*-adenosylmethionine from methionine and ATP, the key methyl donor in cellular processes
*MAT2B*	5q34 (16,495 bp)	ENSG00000038274	The protein encoded in this gene belongs to the methionine adenosyl-transferase (MAT) family, which catalyzes the biosynthesis of *S*-adenosylmethionine
*GNMT*	6p21.1 (34,401 bp)	ENSG00000124713	The enzyme codified if the glycine-N-methyltransferase acts in the cytoplasmatic synthesis of *S*-adenosyl-homocysteine from glycine and *S*-adenosyl-methionine
*AHCY*	20q11.22 (73,856 bp)	ENSG00000101444	The *S*-adenosyl-homocysteine hydrolase regulates the *S*-adenosyl-homocysteine concentration through its hydrolyzation
*BHMT*	5q14.1 (20,480 bp)	ENSG00000145692	It gives rise to the cytosolic enzyme betaine-homocysteine-*S*-methyltransferase, which catalyzes the conversion of betaine and homocysteine to dimethylglycine and methionine
*CHDH*	3p21.1 (34,085 bp)	ENSG00000016391	The protein codified is the choline dehydrogenase, which acts in the synthesis of betaine from choline at mitochondria
*DNMT1*	19p13.2 (94,945 bp)	ENSG00000130816	This gene codes for the enzyme DNA methyltransferase 1, which is responsible for transferring methyl groups to the cytosine nucleotides of genomic DNA. It is the most active enzyme in maintaining the DNA methylation patterns
*DNMT3A*	2p23.3 (114,736 pb)	ENSG00000119772	It codes for the enzyme DNA methyltransferase 3A, which is responsible for de novo methylations of the CpG sites in genomic DNA
*DNMT3B*	20q11.21 (46,972 pb)	ENSG00000088305	It codes for the enzyme DNA methyltransferase 3B, which is responsible for de novo methylations of the CpG sites in genomic DNA
*CBS*	21q22.3 (23,753 pb)	ENSG00000160200	The synthesized protein, the cystathionine-β-synthase, is a tetramer that converts homocysteine to cystathionine. It initiates the trans-sulphurization pathway
*CTH*	1p31.1 (28,634 pb)	ENSG00000116761	This gene codes for the cytoplasmic enzyme γ-cystathionine lyase that converts methionine-derived cystathionine to cysteine
*PRMT1*	19q13.33 (13,244 bp)	ENSG00000126457	The enzyme arginine-N-methyltransferase is involved in the regulation of several biological processes by methylating the amino-terminal groups of arginine. High expression of the enzyme is associated with various types of cancer
*ALDH1L1*	3q21.3 (94,433 bp)	ENSG00000144908	The enzyme belongs to the aldehyde dehydrogenase family and catalyzes the conversion of 10-mTHF, NADP+, and water to THF, NADPH, and carbon dioxide
*ALDH1L2*	12q23.3 (87,860 bp)	ENSG00000136010	It is the mitochondrial form of FDH, which plays an essential role in the distribution of one-carbon groups
*AMT*	3p21.31 (5696 bp)	ENSG00000145020	It encodes one of the main components of the glycine cleavage system, which participates in the catalysis of glycine
*ATIC*	2q35 (37,706 bp)	ENSG00000138363	The gene encodes a bifunctional enzyme that catalyzes the last two steps of the de novo purine biosynthesis pathway
*CD320*	19p13.2 (6232 bp)	ENSG00000167775	This gene encodes the transcobalamin receptor, which is expressed in the cell surface. It mediates the cellular uptake of transcobalamin-bound cobalamin (vitamin B12)
*CUBN*	10p13 (305,846 bp)	ENSG00000107611	This gene encodes for the cubilin. This protein acts as a receptor for intrinsic factor–vitamin B12 complexes. The protein is located within the epithelium of intestine and kidney
*DMGDH*	5q14.1 (72,111 bp)	ENSG00000132837	The gene encodes an enzyme involved in the catabolism of choline, catalyzing the oxidative demethylation of dimethylglycine to form sarcosine at the mitochondria
*FPGS*	9q34.11 (11,224 bp)	ENSG00000136877	The resulting enzyme is the folylpolyglutamate synthetase. It has a central role in establishing and maintaining both cytosolic and mitochondrial folylpolyglutamate levels
*FTCD*	21q22.3 (19,318 bp)	ENSG00000281775	This gene encodes a bifunctional enzyme that channels one-carbon units from the histidine degradation pathway to the folate pool
*GART*	21q22.11 (38,961 bp)	ENSG00000262473	The result of this gene translation is a trifunctional polypeptide that is required for the de novo purine biosynthesis
*CBLIF* *(GIF)*	11q12.1 (16,227 bp)	ENSG00000134812	The resulting protein is a member of the cobalamin transport family. The protein is secreted in the parietal cells, and it is required for adequate absorption of vitamin B12
*MMAB*	12q24.11 (19,790 bp)	ENSG00000139428	This gene encodes a protein that catalyzes the final step in the conversion of vitamin B12 into adenosyl-cobalamin
*MTFMT*	15q22.31 (28,128 bp)	ENSG00000103707	The resulting enzyme is the mitochondrial methionyl-TRNA formyltransferase, which catalyzes the formylation of methionyl-tRNA
*SARDH*	9q34.2 (76,396 bp)	ENSG00000123453	This gene codifies for an enzyme localized in the mitochondrial matrix, the sarcosine dehydrogenase. It catalyzes the oxidative demethylation of sarcosine
*TCN1*	11q12.1 (13,690 bp)	ENSG00000134827	This gene encodes a member of the vitamin B12-binding protein family. The protein facilitates the transport of cobalamin into cells
*TCN2*	22q12.2 (20,098 bp)	ENSG00000185339	The resulting protein is a member of the vitamin B12-binding protein family. This plasma protein binds cobalamin and mediates its transport into cells

5-mTHF: 5-methyl-tetrahydrofolate; 10-mTHF: 10-methyl-tetrahydrofolate; 5,10-mTHF: 5,10-methyl-tetrahydrofolate; dTMP: thymidine-5′ monophosphate; bp: base pair; THF: tetrahydrofolate; ATP: adenosine triphosphate; NADP+/NADPH: nicotinamide adenine dinucleotide phosphate; FDH: 10-formyltetrahidrofolate dehydrogenase.

**Table 2:** Single nucleotide polymorphisms located in the genes related to one-carbon metabolism and potentially associated with cancer (GRCh38.p14 genome-built version).

Gene	rsID	Human Genome Variation Society ID	Type of Variant	Nucleotide Change
*GGH* (8q12.3)	rs719235	NC_000008.11:g.63039122C>A	5′ UTR	C>A
*FOLH1* (11p11.12)	rs10839234	NC_000011.10:g.49163972C>T	Intronic	C>T
*SLC19A1* (21q22.3)	rs9977637	NC_000021.9:g.45494728A>G	3′ UTR	A>G
	rs17004785	NC_000021.9:g.45512704G>A	3′ UTR	G>C
*SLC46A1* (17q11.2)	rs2239910	NC_000017.11:g.28396647C>A	3′ UTR	C>A
*DHFR* (5q14.1)	rs1650697	NC_000005.10:g.80654962A>G	Missense	A>G
*TYMS* (18p11.32)	rs2124616	NC_000018.10:g.661917G>A	Intronic	G>A
	rs11664283	NC_000018.10:g.650968G>A	NCEV ^a^	G>A
*MTHFD1* (14q23.3)	rs2236225	NC_000014.9:g.64442127G>A	Missense	G>A
*MTHFD1L* (6q25.1)	rs803446	NC_000006.12:g.150944078G>A	Intronic	G>A
	rs12660161	NC_000006.12:g.151140796G>A	Intronic	G>A
*MTHFD2* (2p13.1)	rs12469365	NC_000002.12:g.74189113G>A	Intronic	G>A
*MTHFD2L* (4q13.3)	rs7683181	NC_000004.12:g.74237959C>T	Intronic	C>T
	rs7686861	NC_000004.12:g.74132767C>T	Intronic	C>T
*SHMT1* (17p11.2)	rs2168781	NC_000017.11:g.18337432C>G	Intronic	C>G
*MTHFR* (1p36.22)	rs1801131	NC_000001.11:g.11794419T>G	Missense	T>G
*MTHFS* (15q25.1)	rs4778734	NC_000015.10:g.79918509A>G	Intronic	A>G
*MTR* (1q43)	rs1805087	NC_000001.11:g.236885200A>G	Missense	A>G
*MTRR* (5p15.31)	rs1801394	NC_000005.10:g.7870860A>G	Missense	A>G
*MAT1A* (10q22.3)	rs10887718	NC_000010.11:g.80282868C>T	Intronic	C>T
*MAT2A* (2p11.2)	rs2028900	NC_000002.12:g.85540612C>T	Intronic	C>T
*GNMT* (6p21.1)	rs10948059	NC_000006.12:g.42960723C>T	RRV	C>T
*AHCY* (20q11.22)	rs6087571	NC_000020.11:g.34324285A>G	RRV	A>G
*BHMT* (5q14.1)	rs3733890	NC_000005.10:g.79126136G>A	Missense	A>G
*CHDH* (3p21.1)	rs6801605	NC_000003.12:g.53842191A>G	Intronic	A>G
*DNMT1* (19p13.2)	rs2228611	NC_000019.10:g.10156401T>A	SV	T>A
*DNMT3A* (2p23.3)	rs11890065	NC_000002.12:g.25264508C>T	Intronic	C>T
	rs7581217	NC_000002.12:g.25302075T>C	Intronic	T>C
	rs752208	NC_000002.12:g.25232520G>T	3′ UTR	G>T
*DNMT3B* (20q11.21)	rs6141813	NC_000020.11:g.32778437A>G	Intronic	A>G
*CTH* (1p31.1)	rs672203	NC_000001.11:g.70421416A>G	Intronic	A>G
	rs1021737	NC_000001.11:g.70439117G>T	Missense	G>T
*PRMT1* (19q13.33)	rs10415880	NC_000019.10:g.49685899G>A	NCEV ^a^	G>A
*ALDH1L1* (3q21.3)	rs6792028	NC_000003.12:g.126117830G>C	Intronic	G>C
*ALDH1L2* (12q23.3)	rs7954946	NC_000012.12:g.105070258C>T	Intronic	C>T
*CD320* (19p13.2)	rs2232775	NC_000019.10:g.8308268T>C	Missense	T>C
*CUBN* (10p13)	rs1801222	NC_000010.11:g.17114152A>G	Missense	A>G
	rs61841503	NC_000010.11:g.16977560A>G	Intronic	A>G
	rs796667	NC_000010.11:g.16904820G>T	Intronic	G>T
*DMGDH* (5q14.1)	rs4512118	NC_000005.10:g.79056268C>G	Intronic	C>G
*FTCD* (21q22.3)	rs725976	NC_000021.9:g.46137966G>A	Intronic	G>A
*CBLIF (GIF)* (11q12.1)	rs7117509	NC_000011.10:g.59822371A>G	RRV	A>G
*MMAB* (12q24.11)	rs9593	NC_000012.12:g.109557065A>T	Missense	A>T
*SARDH* (9q34.2)	rs2519125	NC_000009.12:g.133670195A>G	Intronic	A>G
	rs2073817	NC_000009.12:g.133694338C>T	Missense	C>T
	rs476835	NC_000009.12:g.133738370C>G	Intronic	C>G
*TCN1* (11q12.1)	rs34324219	NC_000011.10:g.59855905C>A	Missense	C>A
*TCN2* (22q12.2)	rs4820023	NC_000022.11:g.30634794C>T	TFBS	C>T

Information on the genes to which these variants belong, the chromosomic location, the type of genetic variant, and the nucleotide change. 3′ UTR: 3′ untranslated region; 5′ UTR: 5′ untranslated region; NCEV: non-coding exonic variant; RRV: regulating region variant; SV: synonymous variant; TFBS: transcription factor binding site. ^a^ The NCEV polymorphism is not located in 3′ nor 5′ UTRs.

## Data Availability

Data is contained within the article and Supplementary Materials.

## Supplementary Materials

The following supporting information can be downloaded at https://www.mdpi.com/article/10.3390/ijms25158175/s1.
