# Supplementary material for: Molecular Mechanisms Linking Genes and Vitamins of the Complex B Related to One-Carbon Metabolism in Breast Cancer: An In Silico Functional Database Study
Source: Int J Mol Sci. 2024 Jul 26;25(15):8175. doi: 10.3390/ijms25158175 (PMC11311893; doi:10.3390/ijms25158175)
Supplement: Supplementary file 1 [file ijms-25-08175-s001.zip › Table S1.pdf]

Table S1. Influence of vitamins B2, B6, B9, and B12 on breast cancer

1st author (Year)	PMID	Study design	N	Nutrient	Association with breast cancer
Zeng J. (2020)	32241696	Systematic review and meta-analysis	27 studies (49,707 cases /1,224,353 controls)	Folate	High folate intake reduces global breast cancer risk (RR = 0.95, 95%CI: 0.92-0.99, *p* = 0.018). High folate serum levels reduce global breast cancer risk (RR = 0.89, 95%CI: 0.78-0.99, *p* = 0.039). Folate intake reduces ER-/PR- breast cancer risk (RR = 0.83, 95%CI: 0.69-0.98, *p* = 0.031).
				B6	Serum and dietary intake of B6 is not associated with global breast cancer risk. B6 intake decreases ER-/PR- breast cancer risk (RR = 0.76, 95%CI: 0.57-0.99, *p* = 0.048).
				B2	B2 dietary intake reduces global breast cancer risk (RR = 0.93, 95%CI: 0.88-0.98, *p* = 0.014). There was no association between B2 serum levels and global breast cancer risk. B2 intake reduces ER+/PR+ breast cancer risk (RR = 0.78, 95%CI: 0.65-0.94, *p* = 0.011).
				B12	There was no association between B12 serum levels or dietary intake with global breast cancer risk neither breast cancer subtypes.
Hatami M. (2020)	30758268	Case-control	151 cases/154 controls	Folate	Higher dietary folate intake reduces global breast cancer risk (OR = 0.09, 95%CI: 0.04-0.22, *p* < 0.001). Higher total folate intake (dietary and supplementary) reduces global breast cancer risk (OR = 0.09, 95%CI: 0.04-0.21, *p* < 0.001). Higher folate dietary folate intake reduces the risk of developing ER+ breast cancer (OR = 0.09, 95%CI: 0.0.04-0.24, *p* < 0.001).
				B2	Higher dietary B2 intake reduces global breast cancer risk (OR = 0.14, 95%CI: 0.06-0.34, *p* < 0.001). Higher total B2 intake (dietary and supplementary) reduces global breast cancer risk (OR = 0.07, 95%CI: 0.07-0.39, *p* < 0.001). Higher B2 dietary folate intake reduces the risk of developing ER+ breast cancer (OR = 0.10, 95%CI: 0.04-0.27, *p* < 0.001).
				B6	Higher dietary B6 intake reduces global breast cancer risk (OR = 0.08, 95%CI: 0.03-0.21, *p* < 0.001). Higher total B6 intake (dietary and supplementary) reduces global breast cancer risk (OR = 0.11, 95%CI: 0.05-0.27, *p* < 0.001).

					Higher B6 dietary folate intake reduces the risk of developing ER+ breast cancer (OR = 0.06, 95%CI: 0.02-0.18, $p < 0.001$).
				B12	Higher dietary B12 intake reduces global breast cancer risk (OR = 0.31, 95%CI: 0.15-0.65, $p = 0.002$).<br>Higher total B12 intake (dietary and supplementary) reduces global breast cancer risk (OR = 0.20, 95%CI: 0.09-0.43, $p < 0.001$).<br>Higher B12 dietary folate intake reduces the risk of developing ER+ breast cancer (OR = 0.29, 95%CI: 0.13-0.66, $p < 0.001$).
Arthur R.S. (2019)	30955365	Case-cohort	922 cases/3,088 subcohort	Folate	There was no association between folate dietary intake and breast cancer risk.
				B2	There was no association between B2 dietary intake and overall breast cancer.<br>Higher dietary B2 intake reduces premenopausal breast cancer development (OR = 0.58, 95CI: 0.36-0.91, $p = 0.04$).
				B6	There was no association between B2 dietary intake and overall breast cancer.<br>Higher dietary B2 intake reduces premenopausal breast cancer development (OR = 0.59, 95CI: 0.38-0.93, $p = 0.03$).
				B12	There was no association between folate dietary intake and breast cancer risk.
Yu L. (2017)	28035488	Systematic review and meta-analysis	10 studies (12,268 cases/194,530 controls	B2	Higher dietary intake of B2 reduces overall breast cancer risk (RR = 0.85, 95%CI: 0.76-0.95, $p < 0.05$).<br>The increment of 1 mg/day of B2 was inversely related to the risk of breast cancer (RR = 0.94, 95%CI: 0.90-0.99).
Egnell M. (2017)	28505069	Cohort	462 cases/27,391 controls	B2	There was no association B2 between dietary, supplemental or total intake and breast cancer risk.
				B6	Higher dietary intake of B6 reduces the risk of developing breast cancer (HR = 0.74, 95%CI: 0.55-0.99, $p = 0.05$).<br>Higher supplementary intake of B6 reduces the risk of developing breast cancer (HR = 0.61, 95%CI: 0.38-0.98, $p = 0.05$).<br>Higher total intake of B6 reduces the risk of developing breast cancer (HR = 0.67, 95%CI: 0.50-0.91, $p = 0.01$).
Han E. (2024)	38270539	Randomized controlled clinical trial	182 women followed up 7.4 years	Folate	Higher folate dietary intake is inversely associated with the %DBV (Q1 mean = 19.7; Q4 mean = 19.6) ($p = 0.006$).<br>Higher folate dietary intake is inversely associated with the ADBV (Q1 mean = 80.3; Q4 mean = 70.2) ($p = 0.02$).
				B2	There was no association between B2 dietary intake and the ADBV and %DBV.
				B6	There was no association between B6 dietary intake and the ADBV and %DBV.

				B12	There was no association between B12 dietary intake and the ADBV and %DBV.
Wu W. (2013)	23907430	Meta-analysis	5 studies (2,509 cases)	B6	Higher serum levels of B6 decrease overall breast cancer risk (RR = 0.80, 95%CI: 0.66-0.98, p = 0.03) and postmenopausal breast cancer risk (RR = 0.71, 95%CI: 0.57-0.80, p = 0.001). There was no association between dietary B6 intake and the risk of developing breast cancer.
			-	B12	There was no association between dietary or serum levels of vitamin B12 and the risk of breast cancer.
Kim S.J. (2016)	27465373	Cohort	164 *BRCA1*/*BRCA2*-mutation carriers with no previous history of cancer	Folate	Women with high plasma folate concentration presented a higher risk of developing breast cancer (HR: 3.20, 95%CI: 1.03-9.92, *p* = 0.04).
			162 *BRCA1*/*BRCA2*-mutation carriers with no previous history of cancer	B6	There was no association between B6 plasma levels and the risk of developing breast cancer.
			124 *BRCA1*/*BRCA2*-mutation carriers with no previous history of cancer	B12	There was no association between B12 plasma levels and the risk of developing breast cancer.
Houghton S.C. (2019)	30346061	Case-control	1,874 cases/1,874 controls	Folate	There was no association between higher plasma folate concentration and breast cancer risk and did not vary by in situ/invasive, hormone receptors status or molecular subtype
				B2	There was no association between higher plasma B2 concentration and breast cancer risk and did not vary by in situ/invasive, hormone receptors status or molecular subtype
				B6	There was no association between higher plasma B6 concentration and breast cancer risk and did not vary by in situ/invasive, hormone receptors status or molecular subtype
Matejcic M. (2017)	27905104	Case-control	2,491 cases/2,521 controls	Folate	There was no association between plasma folate levels and overall or hormone receptors status and breast cancer risk. There was no association between plasma folate intake and breast cancer risk in women consuming above the median level of alcohol intake.
				B12	There was no association between plasma folate levels and overall or hormone receptors status and breast cancer risk. A marginally positive association between B12 status and breast cancer risk in women consuming above the median of alcohol (OR = 1.26, 95%IC: 1.00-1.58, p = 0.05)
				Folate + B12	B12 status was positively associated with breast cancer risk in women with plasma folate levels below the median (OR = 1.29, 95%IC: 1.02-1.62, *p* = 0.03).

OR = odds ratio, HR = hazard ratio, CI: confidence interval, ER: estrogen receptor; PR: progesterone receptor; ADBV: absolute dense breast volume; %DBV: percentage of dense breast volume
