# Supplementary material for: Molecular Mechanisms Linking Genes and Vitamins of the Complex B Related to One-Carbon Metabolism in Breast Cancer: An In Silico Functional Database Study
Source: Int J Mol Sci. 2024 Jul 26;25(15):8175. doi: 10.3390/ijms25158175 (PMC11311893; doi:10.3390/ijms25158175)
Supplement: Supplementary file 1 [file ijms-25-08175-s001.zip › Table S2.pdf]

Table S2. Association value and cancer-related outcomes of the selected genetic variants linked to the one-carbon metabolism

Gene	SNP rsID	*p*-value	Associated allele	Amino acid [Codon]	Cancer-related outcome	Study reference	Reference database	Population
GGH	rs719235	$1x10^{-77}$	A	N/A	Lower blood protein levels	GCST90090663	GWAS Catalog	Caucasian from Europe
		$1x10^{-177}$	A		Lower blood protein levels	GCST90247726	GWAS Catalog	Caucasian from Europe
FOLH1	rs10839234	$2x10^{-57}$	T	N/A	Higher blood levels of the metabolite NAAG	GCST90139445	GWAS Catalog	Caucasian from Europe
DHFR	rs1650697	$1x10^{-27}$	A	N/A	Brain cortex thickness	GCST90095131	GWAS Catalog	Caucasian from Europe
TYMS	rs2124616	$1x10^{-11}$	A	N/A	Lower corpuscular volume of hemoglobin	GCST90002390	GWAS Catalog	Caucasian from Europe
		$2x10^{-8}$	A	N/A	Shorter telomeres length in leucocytes	GCST009856	GWAS Catalog	Caucasian from Europe
MTHFD1	rs2236225	0.004	A	Arg [CGC] > Gln [CAG]	Increased risk of developing breast cancer	38433849	PubMed	Caucasian
MTHFD2	rs12469365	$1x10^{-23}$	A	N/A	Lower protein levels in the mitochondrion	GCST90248532	GWAS Catalog	Caucasian from Europe
SHMT1	rs1979277	0.020	C	N/A	Lower risk of developing ER+ breast cancer	25598430	PubMed	Mixed population (Caucasian and African)
MTHFR	rs1801131	$4x10^{-9}$	A	Glu [GAA] > Ala [GCA]	Later menopause age and lower homocysteine levels	GCST90020026	GWAS Catalog	Caucasian from Europe
MTR	rs1805087	0.010	G	Asp [GAC] > Gly [GGC]	Reduces the risk of developing breast cancer	31549463[a]	PubMed	Caucasian
MTRR	rs1801394	0.040	G	Ile [ATA] > Met [ATG]	Increases the risk of developing breast cancer	31549463[a]	PubMed	Asian (from the south)
		0.020	G		Increases the risk of developing breast cancer	32744066[a]	PubMed	Asian (from India)
MAT1A	rs10887718	$1x10^{-10}$	T	N/A	Decreases vitamin D serum levels	GCST010144	GWAS Catalog	Caucasian from Europe
		$5x10^{-9}$	C		Increases vitamin D serum levels	GCST90000616	GWAS Catalog	Caucasian from Europe
GNMT	rs10948059	$1x10^{-9}$	C	N/A	Lower HDL cholesterol levels in blood	GCST007140	GWAS Catalog	Mixed population

								(mainly Caucasians)
AHCY	rs6087571	2x10^{-14}	C	N/A	Higher BMI adjusted by the hip circumference	GCST012227	GWAS Catalog	Caucasian from Europe
BHMT	rs3733890	2x10^{-9}	A	Arg [CGA] > Gln [CAA]	Decreases blood HDL cholesterol levels	GCST90019510	GWAS Catalog	Caucasian from Europe
		4x10^{-11}	G		Higher HDL cholesterol levels in blood	GCST010242	GWAS Catalog	Mixed population (mainly Caucasians)
CHDH	rs6801605	2x10^{-18}	A	N/A	Lower expression of IL17B receptor	GCST009569	GWAS Catalog	Caucasian from Europe
DNMT1	rs2228611	0.030	A	N/A	Decreases the risk of developing breast cancer	35046699	PubMed	Asian
		0.016	G		Increases the risk of developing breast cancer	28473984[a]	PubMed	Mixed population (Caucasian and Asian)
DNMT3A	rs11890065	1x10^{-11}	-	N/A	Related to BMI adjusted by the hip circumference	GCST009867	GWAS Catalog	Caucasian from Europe
	rs7581217	4x10^{-10}	-	N/A	Related to BMI	GCST009871	GWAS Catalog	Caucasian from Europe
	rs752208	2x10^{-15}	A	N/A	Higher BMI	GCST90179150	GWAS Catalog	Caucasian from Europe
DNMT3B	rs6141813	1x10^{-8}	-	N/A	Decreases the blood levels of glycerol-3-phosphate	GCST009391	GWAS Catalog	Caucasian from Europe
CTH	rs672203	2x10^{-8}	A		Adverse response to breast cancer chemotherapy (alopecia)	GCST002178	GWAS Catalog	Asian
	rs1021737	9x10^{-9}	-	Ser [AGT] > Asn [AAT]	Higher blood triglycerides levels	GCST009391	GWAS Catalog	Caucasian from Europe
PRMT1	rs10415880	1x10^{-30}	A	N/A	Higher height	GCST90245848	GWAS Catalog	Mixed population (mainly Caucasian)
ALDH1L1	rs6792028	3x10^{-11}	G	N/A	Increase glycine levels	GCST90092820	GWAS Catalog	Caucasian from Europe
ALDH1L2	rs7954946	9x10^{-32}	T	N/A	It increases body height	GCST90245848	GWAS Catalog	Mixed population

CD320	rs2232775	$5x10^{-13}$	C	Gln [CAG] > Arg [CCG]	Increase the holo-transcobalamin-2 levels	GCST90237969	GWAS Catalog	African American
CUBN	rs1801222	$2x10^{-13}$	A	Phe [TAT] > Cys [TGT]	Decrease vitamin B12 levels	GCST000483	GWAS Catalog	Caucasian from Europe
		$8x10^{-10}$	A		Increase homocysteine levels	GCST002087	GWAS Catalog	Caucasian from Europe
		$2x10^{-13}$	-		It is associated with the body height	GCST007841	GWAS Catalog	Caucasian from Europe
	rs61841503	$1x10^{-8}$	G	N/A	It is associated with gut microbiota diversity	GCST90016946	GWAS Catalog	Mixed population
	rs796667	$5x10^{-28}$	T	N/A	It decreases vimentin levels	GCST90250168	GWAS Catalog	Caucasian from Europe
		$2x10^{-15}$	T		It decreases alpha-intermexin levels	GCST90246468	GWAS Catalog	Caucasian from Europe
DMGDH	rs4512118	$2x10^{-43}$	C	N/A	Increase serum levels of dimethylglycine	GCST012020	GWAS Catalog	Hispanic/Latin American
FTCD	rs725976	$8x10^{-12}$	-	N/A	It is associated with the waist circumference adjusted for body mass index	GCST009867	GWAS Catalog	Caucasian from Europe
CBLIF	rs7117509	$6x10^{-18}$	G	N/A	Decrease body height	GCST90245848	GWAS Catalog	Mixed population
MAT2A	rs2028900	$3x10^{-38}$	C	N/A	It is associated with prostate carcinoma	GCST011049	GWAS Catalog	Mixed population
		$1x10^{-11}$	T		Decrease basophil count	GCST90002292	GWAS Catalog	Caucasian from Europe
MMAB	rs9593	$4x10^{-14}$	A	Met [AAG] > Arg [AGG]	It is associated with prostate carcinoma	GCST90274713	GWAS Catalog	Mixed population
		$8x10^{-9}$	A		Decrease phospholipid levels in small HDL	GCST90092952	GWAS Catalog	Caucasian from Europe
		$2x10^{-8}$	A		Decrease free cholesterol levels in small HDL	GCST90092948	GWAS Catalog	Caucasian from Europe
MTHFD1L	rs803446	$5x10^{-9}$	-	N/A	It is associated with total PHF-tau measurement	GCST010340	GWAS Catalog	Not specified
	rs12660161	$2x10^{-9}$	A	N/A	Increase the body height	GCST90245844	GWAS Catalog	Hispanic/Latin American

MTHFD2L	rs7683181	1x10^{-16}	C	N/A	It increases the growth-regulated alpha protein levels	GCST90161572	GWAS Catalog	Caucasian from Middle East
	rs7686861	1x10^{-11}	-	N/A	It is associated with white blood cell count	GCST90278647	GWAS Catalog	Asian
MTHFS	rs4778734	5x10^{-30}	G	N/A	Increase eosinophil counts	GCST90018953	GWAS Catalog	Mixed population (European and Asian)
SARDH	rs2519125	1x10^{-16}	G	N/A	It increases sarcosine measurement	GCST90139536	GWAS Catalog	Caucasian from Europe
	rs2073817	2x10^{-19}	T	Arg [CGT] > Pro [CTT]	It increases levels of sarcosine in blood	GCST90300221	GWAS Catalog	Mixed population
	rs476835	8x10^{-29}	G	N/A	It increases levels of sarcosine in blood	GCST90300221	GWAS Catalog	Mixed population
SLC19A1	rs9977637	9x10^{-10}	G	N/A	It decreases total cholesterol levels	GCST90239676	GWAS Catalog	Caucasian from Europe
	rs17004785	5x10^{-73}	C	N/A	It decreases endostatin levels	GCST90247456	GWAS Catalog	Caucasian from Europe
SLC46A1	rs2239910	1x10^{-15}	A	N/A	It decreases 17-beta-hydroxysteroid dehydrogenase 14 levels	GCST90247275	GWAS Catalog	Caucasian from Europe
TCN1	rs34324219	4x10^{-8}	A	Asp [GAT] > Tyr [TAT]	Decrease vitamin B12 levels 0.34 pmol/L	GCST004161	GWAS Catalog	South Asian (from India)
		3x10^{-125}	A		Decrease transcobalamin 1 levels in blood	GCST90249967	GWAS Catalog	Caucasian from Europe
TCN2	rs4820023	1x10^{-120}	T	N/A	Decrease transcobalamin 2 levels in blood	GCST006585	GWAS Catalog	Caucasian from Europe

BMI: body mass index; HDL: high density lipoprotein; NAAG: N-acetyl-aspartyl glutamate; IL17B: interleukin 17 B; N/A means there is no amino acid change associated with the SNP; Arg: arginine; Gln: glutamine; Glu: glutamate; Ala: alanine; Asp: aspartate; Gly: glycine; Ile: isoleucine; Met: methionine; Ser: serine; Asn: asparagine; Phe; phenylalanine; Cys: cysteine; Pro: proline; Arg: arginine; Tyr: tyrosine.

[a]Results from a meta-analysis
