# Supplementary material for: Molecular Mechanisms Linking Genes and Vitamins of the Complex B Related to One-Carbon Metabolism in Breast Cancer: An In Silico Functional Database Study
Source: Int J Mol Sci. 2024 Jul 26;25(15):8175. doi: 10.3390/ijms25158175 (PMC11311893; doi:10.3390/ijms25158175)
Supplement: Supplementary file 1 [file ijms-25-08175-s001.zip › Table S3.pdf]

**Table S3.** General DEG of the one-carbon metabolism related genes in the tissue/cells from GTEx v8.

| Category | GeneSet | N_ genes | N_ overlap | p | adjP | genes |
|---|---|---|---|---|---|---|
| DEG.up | Adipose_Tissue | 1919 | 6 | 0.062557503 | 1 | ENSG00000164933:ENSG00000100714:ENSG00000065911:ENSG00000176974:ENSG00000144908:ENSG00000185339 |
| DEG.up | Adrenal_Gland | 1549 | 4 | 0.19570689 | 1 | ENSG00000076351:ENSG00000144908:ENSG00000132837:ENSG00000139428 |
| DEG.up | Bladder | 843 | 0 | 1 | 1 | |
| DEG.up | Blood | 2296 | 4 | 0.4453333 | 1 | ENSG00000173638:ENSG00000176890:ENSG00000136371:ENSG00000134827 |
| DEG.up | Blood_Vessel | 2703 | 5 | 0.371897966 | 1 | ENSG00000164933:ENSG00000120254:ENSG00000065911:ENSG00000116984:ENSG00000136010 |
| DEG.up | Brain | 3221 | 3 | 0.869202982 | 1 | ENSG00000086205:ENSG00000016391:ENSG00000144908 |
| DEG.up | Breast | 1978 | 8 | 0.007953809 | 0.238614279 | ENSG00000110195:ENSG00000065911:ENSG00000176974:ENSG00000124713:ENSG00000016391:ENSG00000167775:ENSG00000134827:ENSG00000185339 |
| DEG.up | Cervix_Uteri | 3029 | 4 | 0.668695811 | 1 | ENSG00000164933:ENSG00000065911:ENSG00000130816:ENSG00000103707 |
| DEG.up | Colon | 1580 | 0 | 1 | 1 | |
| DEG.up | Esophagus | 1264 | 2 | 0.563797004 | 1 | ENSG00000145692:ENSG00000134827 |
| DEG.up | Fallopian_Tube | 490 | 2 | 0.164002128 | 1 | ENSG00000145020:ENSG00000136877 |
| DEG.up | Heart | 614 | 0 | 1 | 1 | |
| DEG.up | Kidney | 1363 | 11 | 3.62E+08 | 0.000108588 | ENSG00000110195:ENSG00000136371:ENSG00000176974:ENSG00000145692:ENSG00000016391:ENSG00000144908:ENSG00000107611:ENSG00000132837:ENSG00000160282:ENSG00000123453:ENSG00000185339 |
| DEG.up | Liver | 1536 | 17 | 2.60E+02 | 7.79E+04 | ENSG00000137563:ENSG00000086205:ENSG00000076351:ENSG00000100714:ENSG00000136371:ENSG00000176974:ENSG00000151224:ENSG00000124713:ENSG00000145692:ENSG00000016391:ENSG00000160200:ENSG00000116761:ENSG00000144908:ENSG00000132837:ENSG00000160282:ENSG00000139428:ENSG00000123453 |
| DEG.up | Lung | 4262 | 11 | 0.043584728 | 1 | ENSG00000110195:ENSG00000173638:ENSG00000120254:ENSG00000065911:ENSG00000136371:ENSG00000177000:ENSG00000124275:ENSG00000130816:ENSG00000136877:ENSG00000123453:ENSG00000185339 |
| DEG.up | Muscle | 1143 | 1 | 0.82209501 | 1 | ENSG00000144908 |
| DEG.up | Nerve | 5911 | 21 | 3.50E+09 | 0.001050598 | ENSG00000086205:ENSG00000173638:ENSG00000164933:ENSG00000120254:ENSG00000176974:ENSG00000177000:ENSG00000116984:ENSG00000124275:ENSG00000145692:ENSG00000130816:ENSG00000088305:ENSG00000160200:ENSG00000116761:ENSG00000136010:ENSG00000145020:ENS |

| | | | | | | |
|---|---|---|---|---|---|---|
| | | | | | | G00000107611:ENSG00000132837:ENSG00000136877:ENSG00000139428:ENSG00000103707:ENSG00000185339 |
| DEG.up | Ovary | 5714 | 17 | 0.00232858 | 0.069857408 | ENSG00000173638:ENSG00000164933:ENSG00000120254:ENSG00000177000:ENSG00000116984:ENSG00000151224:ENSG00000119772:ENSG00000160200:ENSG00000116761:ENSG00000144908:ENSG00000145020:ENSG00000107611:ENSG00000136877:ENSG00000160282:ENSG00000139428:ENSG00000103707:ENSG00000123453 |
| DEG.up | Pancreas | 747 | 7 | 0.000107286 | 0.003218578 | ENSG00000151224:ENSG00000124713:ENSG00000016391:ENSG00000088305:ENSG00000160200:ENSG00000136010:ENSG00000123453 |
| DEG.up | Pituitary | 5188 | 12 | 0.071264052 | 1 | ENSG00000110195:ENSG00000164933:ENSG00000076351:ENSG00000163738:ENSG00000136371:ENSG00000177000:ENSG00000124713:ENSG00000016391:ENSG00000119772:ENSG00000145020:ENSG00000107611:ENSG00000123453 |
| DEG.up | Prostate | 3333 | 7 | 0.219518842 | 1 | ENSG00000086205:ENSG00000163738:ENSG00000136371:ENSG00000124713:ENSG00000016391:ENSG00000145020:ENSG00000139428 |
| DEG.up | Salivary_Gland | 1899 | 8 | 0.006264732 | 0.187941971 | ENSG00000110195:ENSG00000086205:ENSG00000176974:ENSG00000124713:ENSG00000016391:ENSG00000144908:ENSG00000136010:ENSG00000134827 |
| DEG.up | Skin | 3140 | 7 | 0.178718057 | 1 | ENSG00000137563:ENSG00000176890:ENSG00000120254:ENSG00000182199:ENSG00000151224:ENSG00000088305:ENSG00000136010 |
| DEG.up | Small_Intestine | 2786 | 4 | 0.601197542 | 1 | ENSG00000176890:ENSG00000016391:ENSG00000107611:ENSG00000185339 |
| DEG.up | Spleen | 4308 | 9 | 0.179883286 | 1 | ENSG00000173638:ENSG00000076351:ENSG00000176890:ENSG00000177000:ENSG00000130816:ENSG00000119772:ENSG00000167775:ENSG00000134827:ENSG00000185339 |
| DEG.up | Stomach | 1109 | 4 | 0.081132118 | 1 | ENSG00000110195:ENSG00000124713:ENSG00000016391:ENSG00000134827 |
| DEG.up | Testis | 12379 | 13 | 0.963253657 | 1 | ENSG00000137563:ENSG00000228716:ENSG00000176890:ENSG00000163738:ENSG00000151224:ENSG00000016391:ENSG00000130816:ENSG00000088305:ENSG00000167775:ENSG00000107611:ENSG00000160282:ENSG00000159131:ENSG00000185339 |
| DEG.up | Thyroid | 5722 | 15 | 0.015098732 | 0.452961946 | ENSG00000110195:ENSG00000173638:ENSG00000163738:ENSG00000176974:ENSG00000177000:ENSG00000116984:ENSG00000088305:ENSG00000160200:ENSG00000116761:ENSG00000145020:ENSG00000167775:ENSG00000107611:ENSG00000132837:ENSG00000139428:ENSG00000185339 |
| DEG.up | Uterus | 5589 | 10 | 0.308498297 | 1 | ENSG00000164933:ENSG00000065911:ENSG00000116984:ENSG00000130816:ENSG00000119772:ENSG00000145020:ENSG00000167775:ENSG00000136877:ENSG00000103707:ENSG00000185339 |
| DEG.up | Vagina | 2864 | 2 | 0.933968182 | 1 | ENSG00000137563:ENSG00000134827 |
| DEG.down | Adipose_Tissue | 1601 | 4 | 0.211738487 | 1 | ENSG00000176890:ENSG00000124713:ENSG00000016391:ENSG00000123453 |

| | | | | | | |
|---|---|---|---|---|---|---|
| DEG.down | Adrenal_Gland | 3492 | 9 | 0.067716184 | 1 | ENSG00000137563:ENSG00000228716:ENSG00000176890:ENSG00000065911:ENSG00000176974:ENSG00000116984:ENSG00000124713:ENSG00000130816:ENSG00000136010 |
| DEG.down | Bladder | 105 | 0 | 1 | 1 | |
| DEG.down | Blood | 6212 | 15 | 0.03038118 | 0.9114354 | ENSG00000164933:ENSG00000176974:ENSG00000177000:ENSG00000116984:ENSG00000124275:ENSG00000168906:ENSG00000101444:ENSG00000016391:ENSG00000126457:ENSG00000136010:ENSG00000145020:ENSG00000138363:ENSG00000167775:ENSG00000136877:ENSG00000139428 |
| DEG.down | Blood_Vessel | 1722 | 6 | 0.040528942 | 1 | ENSG00000137563:ENSG00000176890:ENSG00000176974:ENSG00000124713:ENSG00000016391:ENSG00000088305 |
| DEG.down | Brain | 5569 | 16 | 0.00475247 | 0.142574112 | ENSG00000110195:ENSG00000164933:ENSG00000176890:ENSG00000100714:ENSG00000065911:ENSG00000136371:ENSG00000176974:ENSG00000182199:ENSG00000177000:ENSG00000116984:ENSG00000124713:ENSG00000101444:ENSG00000136010:ENSG00000136877:ENSG00000159131:ENSG00000185339 |
| DEG.down | Breast | 1007 | 2 | 0.442260957 | 1 | ENSG00000176890:ENSG00000123453 |
| DEG.down | Cervix_Uteri | 47 | 0 | 1 | 1 | |
| DEG.down | Colon | 1387 | 5 | 0.053359179 | 1 | ENSG00000086205:ENSG00000120254:ENSG00000124713:ENSG00000116761:ENSG00000144908 |
| DEG.down | Esophagus | 1689 | 8 | 0.003092896 | 0.092786879 | ENSG00000110195:ENSG00000086205:ENSG00000173638:ENSG00000120254:ENSG00000124713:ENSG00000016391:ENSG00000136010:ENSG00000160282 |
| DEG.down | Fallopian_Tube | 1 | 0 | 1 | 1 | |
| DEG.down | Heart | 9435 | 29 | 4.77E+08 | 0.00014309 | ENSG00000137563:ENSG00000173638:ENSG00000164933:ENSG00000076351:ENSG00000228716:ENSG00000176890:ENSG00000100714:ENSG00000120254:ENSG00000065911:ENSG00000163738:ENSG00000136371:ENSG00000176974:ENSG00000182199:ENSG00000116984:ENSG00000124275:ENSG00000038274:ENSG00000124713:ENSG00000016391:ENSG00000130816:ENSG00000119772:ENSG00000116761:ENSG00000144908:ENSG00000136010:ENSG00000138363:ENSG00000167775:ENSG00000136877:ENSG00000159131:ENSG00000139428:ENSG00000185339 |
| DEG.down | Kidney | 5433 | 6 | 0.839155238 | 1 | ENSG00000164933:ENSG00000065911:ENSG00000124713:ENSG00000130816:ENSG00000119772:ENSG00000159131 |
| DEG.down | Liver | 8059 | 15 | 0.192363295 | 1 | ENSG00000164933:ENSG00000176890:ENSG00000120254:ENSG00000065911:ENSG00000177000:ENSG00000116984:ENSG00000038274:ENSG00000130816:ENSG00000119772:ENSG00000126457:ENSG00000138363:ENSG00000167775:ENSG00000159131:ENSG00000103707:ENSG00000185339 |
| DEG.down | Lung | 911 | 1 | 0.74609326 | 1 | ENSG00000144908 |

| | | | | | | |
|---|---|---|---|---|---|---|
| DEG.down | Muscle | 6908 | 14 | 0.12490341 | 1 | ENSG00000137563:ENSG00000164933:ENSG00000076351:ENSG00000136371:ENSG00000176974:ENSG00000177000:ENSG00000038274:ENSG00000124713:ENSG00000016391:ENSG00000130816:ENSG00000116761:ENSG00000145020:ENSG00000167775:ENSG00000185339 |
| DEG.down | Nerve | 909 | 1 | 0.74531649 | 1 | ENSG00000176890 |
| DEG.down | Ovary | 1539 | 4 | 0.192671012 | 1 | ENSG00000137563:ENSG00000086205:ENSG00000016391:ENSG00000136010 |
| DEG.down | Pancreas | 9668 | 23 | 0.005782147 | 0.173464422 | ENSG00000110195:ENSG00000137563:ENSG00000173638:ENSG00000164933:ENSG00000176890:ENSG00000100714:ENSG00000120254:ENSG00000163738:ENSG00000176974:ENSG00000177000:ENSG00000116984:ENSG00000124275:ENSG00000038274:ENSG00000130816:ENSG00000119772:ENSG00000126457:ENSG00000138363:ENSG00000167775:ENSG00000136877:ENSG00000159131:ENSG00000139428:ENSG00000103707:ENSG00000185339 |
| DEG.down | Pituitary | 1937 | 4 | 0.322499367 | 1 | ENSG00000137563:ENSG00000100714:ENSG00000176974:ENSG00000144908 |
| DEG.down | Prostate | 744 | 3 | 0.097665967 | 1 | ENSG00000137563:ENSG00000100714:ENSG00000120254 |
| DEG.down | Salivary_Gland | 1870 | 5 | 0.141581458 | 1 | ENSG00000173638:ENSG00000100714:ENSG00000120254:ENSG00000177000:ENSG00000116761 |
| DEG.down | Skin | 2151 | 9 | 0.003837006 | 0.115110186 | ENSG00000173638:ENSG00000076351:ENSG00000136371:ENSG00000124713:ENSG00000016391:ENSG00000119772:ENSG00000144908:ENSG00000134827:ENSG00000185339 |
| DEG.down | Small_Intestine | 1384 | 4 | 0.147847336 | 1 | ENSG00000120254:ENSG00000116761:ENSG00000144908:ENSG00000136010 |
| DEG.down | Spleen | 2075 | 3 | 0.601474449 | 1 | ENSG00000151224:ENSG00000116761:ENSG00000136010 |
| DEG.down | Stomach | 2622 | 5 | 0.347397377 | 1 | ENSG00000137563:ENSG00000228716:ENSG00000120254:ENSG00000144908:ENSG00000132837 |
| DEG.down | Testis | 2951 | 6 | 0.269670583 | 1 | ENSG00000086205:ENSG00000076351:ENSG00000100714:ENSG00000182199:ENSG00000124713:ENSG00000136010 |
| DEG.down | Thyroid | 1042 | 3 | 0.200064338 | 1 | ENSG00000137563:ENSG00000086205:ENSG00000144908 |
| DEG.down | Uterus | 846 | 0 | 1 | 1 | |
| DEG.down | Vagina | 649 | 4 | 0.015424943 | 0.462748297 | ENSG00000120254:ENSG00000124713:ENSG00000016391:ENSG00000144908 |
| DEG.twoside | Adipose_Tissue | 3520 | 10 | 0.030854381 | 0.925631427 | ENSG00000164933:ENSG00000176890:ENSG00000100714:ENSG00000065911:ENSG00000176974:ENSG00000124713:ENSG00000016391:ENSG00000144908:ENSG00000123453:ENSG00000185339 |
| DEG.twoside | Adrenal_Gland | 5041 | 13 | 0.02794819 | 0.838445704 | ENSG00000137563:ENSG00000076351:ENSG00000228716:ENSG00000176890:ENSG00000065911:ENSG00000176974:ENSG00000116984:ENSG00000124713:ENSG00000130816:ENSG00000144908:ENSG00000136010:ENSG00000132837:ENSG00000139428 |

| | | | | | | |
|---|---|---|---|---|---|---|
| DEG.twoside | Bladder | 948 | 0 | 1 | 1 | |
| DEG.twoside | Blood | 8508 | 19 | 0.028902867 | 0.867086024 | ENSG00000173638:ENSG00000164933:ENSG00000176890:ENSG00000136371:ENSG00000176974:ENSG00000177000:ENSG00000116984:ENSG00000124275:ENSG00000168906:ENSG00000101444:ENSG00000016391:ENSG00000126457:ENSG00000136010:ENSG00000145020:ENSG00000138363:ENSG00000167775:ENSG00000136877:ENSG00000139428:ENSG00000134827 |
| DEG.twoside | Blood_Vessel | 4425 | 11 | 0.054897187 | 1 | ENSG00000137563:ENSG00000164933:ENSG00000176890:ENSG00000120254:ENSG00000065911:ENSG00000176974:ENSG00000116984:ENSG00000124713:ENSG00000016391:ENSG00000088305:ENSG00000136010 |
| DEG.twoside | Brain | 8790 | 19 | 0.039744713 | 1 | ENSG00000110195:ENSG00000086205:ENSG00000164933:ENSG00000176890:ENSG00000100714:ENSG00000065911:ENSG00000136371:ENSG00000176974:ENSG00000182199:ENSG00000177000:ENSG00000116984:ENSG00000124713:ENSG00000101444:ENSG00000016391:ENSG00000144908:ENSG00000136010:ENSG00000136877:ENSG00000159131:ENSG00000185339 |
| DEG.twoside | Breast | 2985 | 10 | 0.010747795 | 0.322433864 | ENSG00000110195:ENSG00000176890:ENSG00000065911:ENSG00000176974:ENSG00000124713:ENSG00000016391:ENSG00000167775:ENSG00000123453:ENSG00000134827:ENSG00000185339 |
| DEG.twoside | Cervix_Uteri | 3076 | 4 | 0.680861551 | 1 | ENSG00000164933:ENSG00000065911:ENSG00000130816:ENSG00000103707 |
| DEG.twoside | Colon | 2967 | 5 | 0.451734997 | 1 | ENSG00000086205:ENSG00000120254:ENSG00000124713:ENSG00000116761:ENSG00000144908 |
| DEG.twoside | Esophagus | 2953 | 10 | 0.009997194 | 0.29991581 | ENSG00000110195:ENSG00000086205:ENSG00000173638:ENSG00000120254:ENSG00000124713:ENSG00000145692:ENSG00000016391:ENSG00000136010:ENSG00000160282:ENSG00000134827 |
| DEG.twoside | Fallopian_Tube | 491 | 2 | 0.164525089 | 1 | ENSG00000145020:ENSG00000136877 |
| DEG.twoside | Heart | 10049 | 29 | 1.88E+09 | 0.000563519 | ENSG00000137563:ENSG00000173638:ENSG00000164933:ENSG00000076351:ENSG00000228716:ENSG00000176890:ENSG00000100714:ENSG00000120254:ENSG00000065911:ENSG00000163738:ENSG00000136371:ENSG00000176974:ENSG00000182199:ENSG00000116984:ENSG00000124275:ENSG00000038274:ENSG00000124713:ENSG00000016391:ENSG00000130816:ENSG00000119772:ENSG00000116761:ENSG00000144908:ENSG00000136010:ENSG00000138363:ENSG00000167775:ENSG00000136877:ENSG00000159131:ENSG00000139428:ENSG00000185339 |
| DEG.twoside | Kidney | 6796 | 17 | 0.014274736 | 0.428242088 | ENSG00000110195:ENSG00000164933:ENSG00000065911:ENSG00000136371:ENSG00000176974:ENSG00000124713:ENSG00000145692:ENSG00000016391:ENSG00000130816:ENSG00000119772:ENSG00000144908:ENSG00000107611:ENSG00000132837:ENSG00000160282:ENSG00000159131:ENSG00000123453:ENSG00000185339 |

| | | | | | | |
|---|---|---|---|---|---|---|
| DEG.twoside | Liver | 9595 | 32 | 7.77E+06 | 2.33E+08 | ENSG00000137563:ENSG00000086205:ENSG00000164933:ENSG00000076351:ENSG00000176890:ENSG00000100714:ENSG00000120254:ENSG00000065911:ENSG00000136371:ENSG00000176974:ENSG00000177000:ENSG00000116984:ENSG00000151224:ENSG00000038274:ENSG00000124713:ENSG00000145692:ENSG00000016391:ENSG00000130816:ENSG00000119772:ENSG00000160200:ENSG00000116761:ENSG00000126457:ENSG00000144908:ENSG00000138363:ENSG00000167775:ENSG00000132837:ENSG00000160282:ENSG00000159131:ENSG00000139428:ENSG00000103707:ENSG00000123453:ENSG00000185339 |
| DEG.twoside | Lung | 5173 | 12 | 0.069996351 | 1 | ENSG00000110195:ENSG00000173638:ENSG00000120254:ENSG00000065911:ENSG00000136371:ENSG00000177000:ENSG00000124275:ENSG00000130816:ENSG00000144908:ENSG00000136877:ENSG00000123453:ENSG00000185339 |
| DEG.twoside | Muscle | 8051 | 15 | 0.191243835 | 1 | ENSG00000137563:ENSG00000164933:ENSG00000076351:ENSG00000136371:ENSG00000176974:ENSG00000177000:ENSG00000038274:ENSG00000124713:ENSG00000016391:ENSG00000130816:ENSG00000116761:ENSG00000144908:ENSG00000145020:ENSG00000167775:ENSG00000185339 |
| DEG.twoside | Nerve | 6820 | 22 | 9.33E+09 | 0.002798213 | ENSG00000086205:ENSG00000173638:ENSG00000164933:ENSG00000176890:ENSG00000120254:ENSG00000176974:ENSG00000177000:ENSG00000116984:ENSG00000124275:ENSG00000145692:ENSG00000130816:ENSG00000088305:ENSG00000160200:ENSG00000116761:ENSG00000136010:ENSG00000145020:ENSG00000107611:ENSG00000132837:ENSG00000136877:ENSG00000139428:ENSG00000103707:ENSG00000185339 |
| DEG.twoside | Ovary | 7253 | 21 | 0.000728773 | 0.021863204 | ENSG00000137563:ENSG00000086205:ENSG00000173638:ENSG00000164933:ENSG00000120254:ENSG00000177000:ENSG00000116984:ENSG00000151224:ENSG00000016391:ENSG00000119772:ENSG00000160200:ENSG00000116761:ENSG00000144908:ENSG00000136010:ENSG00000145020:ENSG00000107611:ENSG00000136877:ENSG00000160282:ENSG00000139428:ENSG00000103707:ENSG00000123453 |
| DEG.twoside | Pancreas | 10415 | 30 | 1.11E+09 | 0.000332498 | ENSG00000110195:ENSG00000137563:ENSG00000173638:ENSG00000164933:ENSG00000176890:ENSG00000100714:ENSG00000120254:ENSG00000163738:ENSG00000176974:ENSG00000177000:ENSG00000116984:ENSG00000124275:ENSG00000151224:ENSG00000038274:ENSG00000124713:ENSG00000016391:ENSG00000130816:ENSG00000119772:ENSG00000088305:ENSG00000160200:ENSG00000126457:ENSG00000136010:ENSG00000138363:ENSG00000167775:ENSG00000136877:ENSG00000159131:ENSG00000139428:ENSG00000103707:ENSG00000123453:ENSG00000185339 |
| DEG.twoside | Pituitary | 7125 | 16 | 0.045911775 | 1 | ENSG00000110195:ENSG00000137563:ENSG00000164933:ENSG00000076351:ENSG00000100714:ENSG00000163738:ENSG00000136371:ENSG00000176974:ENSG00000177000:ENSG00000124713:ENSG00000016391:ENSG00000119772:ENSG00000144908:ENSG00000145020:ENSG00000107611:ENSG00000123453 |

| | | | | | | |
|---|---|---|---|---|---|---|
| DEG.twoside | Prostate | 4077 | 10 | 0.071940244 | 1 | ENSG00000137563:ENSG00000086205:ENSG00000100714:ENSG00000120254:ENSG00000163738:ENSG00000136371:ENSG00000124713:ENSG00000016391:ENSG00000145020:ENSG00000139428 |
| DEG.twoside | Salivary_Gland | 3769 | 13 | 0.002550135 | 0.076504047 | ENSG00000110195:ENSG00000086205:ENSG00000173638:ENSG00000100714:ENSG00000120254:ENSG00000176974:ENSG00000177000:ENSG00000124713:ENSG00000016391:ENSG00000116761:ENSG00000144908:ENSG00000136010:ENSG00000134827 |
| DEG.twoside | Skin | 5291 | 16 | 0.002804988 | 0.084149649 | ENSG00000137563:ENSG00000173638:ENSG00000076351:ENSG00000176890:ENSG00000120254:ENSG00000136371:ENSG00000182199:ENSG00000151224:ENSG00000124713:ENSG00000016391:ENSG00000119772:ENSG00000088305:ENSG00000144908:ENSG00000136010:ENSG00000134827:ENSG00000185339 |
| DEG.twoside | Small_Intestine | 4170 | 8 | 0.271751863 | 1 | ENSG00000176890:ENSG00000120254:ENSG00000016391:ENSG00000116761:ENSG00000144908:ENSG00000136010:ENSG00000107611:ENSG00000185339 |
| DEG.twoside | Spleen | 6383 | 12 | 0.222952752 | 1 | ENSG00000173638:ENSG00000076351:ENSG00000176890:ENSG00000177000:ENSG00000151224:ENSG00000130816:ENSG00000119772:ENSG00000116761:ENSG00000136010:ENSG00000167775:ENSG00000134827:ENSG00000185339 |
| DEG.twoside | Stomach | 3731 | 9 | 0.094051552 | 1 | ENSG00000110195:ENSG00000137563:ENSG00000228716:ENSG00000120254:ENSG00000124713:ENSG00000016391:ENSG00000144908:ENSG00000132837:ENSG00000134827 |
| DEG.twoside | Testis | 15330 | 19 | 0.893270149 | 1 | ENSG00000137563:ENSG00000086205:ENSG00000076351:ENSG00000228716:ENSG00000176890:ENSG00000100714:ENSG00000163738:ENSG00000182199:ENSG00000151224:ENSG00000124713:ENSG00000016391:ENSG00000130816:ENSG00000088305:ENSG00000136010:ENSG00000167775:ENSG00000107611:ENSG00000160282:ENSG00000159131:ENSG00000185339 |
| DEG.twoside | Thyroid | 6764 | 18 | 0.00577323 | 0.173196889 | ENSG00000110195:ENSG00000137563:ENSG00000086205:ENSG00000173638:ENSG00000163738:ENSG00000176974:ENSG00000177000:ENSG00000116984:ENSG00000088305:ENSG00000160200:ENSG00000116761:ENSG00000144908:ENSG00000145020:ENSG00000167775:ENSG00000107611:ENSG00000132837:ENSG00000139428:ENSG00000185339 |
| DEG.twoside | Uterus | 6435 | 10 | 0.490443488 | 1 | ENSG00000164933:ENSG00000065911:ENSG00000116984:ENSG00000130816:ENSG00000119772:ENSG00000145020:ENSG00000167775:ENSG00000136877:ENSG00000103707:ENSG00000185339 |
| DEG.twoside | Vagina | 3513 | 6 | 0.421853041 | 1 | ENSG00000137563:ENSG00000120254:ENSG00000124713:ENSG00000016391:ENSG00000144908:ENSG00000134827 |
