# Supplementary material for: Molecular Mechanisms Linking Genes and Vitamins of the Complex B Related to One-Carbon Metabolism in Breast Cancer: An In Silico Functional Database Study
Source: Int J Mol Sci. 2024 Jul 26;25(15):8175. doi: 10.3390/ijms25158175 (PMC11311893; doi:10.3390/ijms25158175)
Supplement: Supplementary file 1 [file ijms-25-08175-s001.zip › Table S4.pdf]

Table S4. Biochemical and genetic perturbation gene sets (MsigDB).

GeneSet	N	n	P-value	adjusted P	genes
SOGA_COLORECTAL_CANCER_MYC_UP	79	8	1.69e-12	5.77e-9	MTR, MTHFD2, ATIC, SLC25A32, SHMT2, TYMS, AHCY, GART
LEE_BMP2_TARGETS_DN	854	13	2.93e-9	4.99e-6	MTHFD2, MAT2A, ATIC, MTRR, BHMT, DHFR, SLC25A32, FPGS, SHMT2, MTHFD1, DNMT1, PRMT1, GART
SHETH_LIVER_CANCER_VS_TXNIP_LOSS_PAM4	278	8	3.96e-8	4.49e-5	CTH, DMGDH, BHMT, GNMT, SARDH, CD320, AHCY, CBS
TARTE_PLASMA_CELL_VS_PLASMABLAST_DN	295	8	6.26e-8	5.33e-5	MTHFD2, ATIC, DHFR, GGH, MTHFD1, TYMS, AHCY, GART
RHEIN_ALL_GLUCOCORTICOID_THERAPY_DN	351	8	2.37e-7	1.61e-4	ATIC, DHFR, GGH, SHMT2, MTHFD1, TYMS, PRMT1, AHCY
BENPORATH_ES_1	368	8	3.39e-7	1.92e-4	DNMT3A, MTHFD2, MAT2A, DHFR, MTHFD1, DNMT3B, GART, CBS
DESERT_PERIPORTAL_HEPATOCELLULAR_CARCINOMA_SUBCLASS_UP	155	6	4.05e-7	1.97e-4	CTH, BHMT, GNMT, FOLH1, CBS, FTCD
DODD_NASOPHARYNGEAL_CARCINOMA_DN	1322	13	4.91e-7	2.09e-4	MTR, MTHFD2, MAT2A, ATIC, MTHFD2L, MTRR, DHFR, SLC25A32, SHMT2, TYMS, PRMT1, AHCY, GART
KINSEY_TARGETS_OF_EWSR1_FLII_FUSION_UP	1233	12	1.67e-6	6.31e-4	CTH, MTHFD2, MAT2A, DHFR, MAT2B, SLC25A32, MTHFD1, MTFMT, TYMS, DNMT1, GART, CBS
KOKKINAKIS_METHIONINE_DEPRIVATION_48HR_DN	58	4	4.04e-6	1.27e-3	CTH, MTFMT, TYMS, DNMT1
DIAZ_CHRONIC_MYELOGENOUS_LEUKEMIA_UP	1345	12	4.11e-6	1.27e-3	CTH, MTR, MTHFD2, ATIC, MAT2B, GGH, SLC25A32, SHMT2, MTHFD1, PRMT1, DNMT3B, GART
FEVR_CTNNB1_TARGETS_DN	526	8	4.87e-6	1.38e-3	MTRR, DHFR, CUBN, MTHFD1, TYMS, DNMT1, PRMT1, GART
HOSHIDA_LIVER_CANCER_SUBCLASS_S3	255	6	7.27e-6	1.90e-3	CTH, AMT, BHMT, GGH, MTHFD1, MTHFS, RP11-38G5.4
DESERT_PERIVENOUS_HEPATOCELLULAR_CARCINOMA_SUBCLASS_UP	150	5	8.39e-6	2.04e-3	MTR, ALDH1L1, GGH, MAT1A, MTHFD1
GROSS_HYPOXIA_VIA_HIF1A_UP	71	4	9.10e-6	2.07e-3	CTH, MAT2A, SHMT2, GART
MOOTHA_MITOCHONDRIA	434	7	1.39e-5	2.82e-3	MTHFD2, AMT, SLC25A32, FPGS, SARDH, SHMT2, MTHFD1
PUJANA_BRCA1_PCC_NETWORK	1518	12	1.41e-5	2.82e-3	MTR, MTHFD2, MAT2A, ATIC, DHFR, GGH, SHMT2, TYMS, DNMT1, PRMT1, AHCY, GART
RODRIGUES_THYROID_CARCINOMA_POORLY_DIFFERENTIATED_UP	616	8	1.53e-5	2.90e-3	MTHFD2, ATIC, DHFR, SLC25A32, TYMS, DNMT1, GART, CBS
CHIANG_LIVER_CANCER_SUBCLASS_PROLIFERATION_DN	174	5	1.72e-5	3.08e-3	CTH, ALDH1L1, BHMT, MAT1A, MTHFD1
CARRILLOREIXACH_HEPATOBLASTOMA_VS_NORMAL_DN	1110	10	2.81e-5	4.78e-3	CTH, MTHFD2L, DMGDH, GNMT, SARDH, MAT1A, FOLH1, MTHFD1, MTHFS, RP11-38G5.4, CBS
PUJANA_CHEK2_PCC_NETWORK	717	8	4.53e-5	7.34e-3	MTHFD2, MAT2A, ATIC, DHFR, SHMT2, TYMS, DNMT1, PRMT1
WHITEFORD_PEDIATRIC_CANCER_MARKERS	112	4	5.50e-5	8.52e-3	DHFR, GGH, TYMS, DNMT1
KOKKINAKIS_METHIONINE_DEPRIVATION_96HR_UP	117	4	6.53e-5	9.66e-3	MTR, BHMT, MAT2B, TYMS
DESERT_STEM_CELL_HEPATOCELLULAR_CARCINOMA_SUBCLASS_UP	236	5	7.37e-5	1.01e-2	MTHFD2, MAT2A, ATIC, DNMT1, PRMT1
BROWN_MYELOID_CELL_DEVELOPMENT_DN	121	4	7.44e-5	1.01e-2	MAT1A, SHMT2, AHCY, GART
CAIRO_HEPATOBLASTOMA_DN	254	5	1.04e-4	1.36e-2	CTH, GNMT, SARDH, MAT1A, CBS

MOOTHA_HUMAN_MITODB_6_2002	413	6	1.08e-4	1.36e-2	MTHFD2, AMT, FPGS, SARDH, SHMT2, MTHFD1
ROSTY_CERVICAL_CANCER_PROLIFERATION_CLUSTER	140	4	1.31e-4	1.59e-2	DHFR, GGH, TYMS, DNMT3B
HELLER_HDAC_TARGETS_DN	279	5	1.61e-4	1.90e-2	CTH, ATIC, SHMT2, TYMS, CBS
BASAKI_YBX1_TARGETS_UP	285	5	1.78e-4	2.02e-2	CTH, DHFR, MTHFD1, TYMS, GART
FISCHER_DREAM_TARGETS	913	8	2.41e-4	2.65e-2	MAT2A, DHFR, GGH, SHMT2, MTHFD1, DNMT1, DNMT3B, GART
SESTO_RESPONSE_TO_UV_C7	67	3	2.62e-4	2.70e-2	ATIC, TCN1, MTHFD1
KOKKINAKIS_METHIONINE_DEPRIVATION_96HR_DN	67	3	2.62e-4	2.70e-2	CTH, MTFMT, DNMT1
DUTERTRE_ESTRADIOL_RESPONSE_24HR_UP	315	5	2.83e-4	2.83e-2	GGH, MTHFD1, TYMS, CD320, DNMT1
YOSHIMURA_MAPK8_TARGETS_UP	1228	9	3.56e-4	3.36e-2	MTHFR, MAT2A, BHMT, DHFR, GNMT, CUBN, MAT1A, TYMS, CBS
GRAHAM_CML_DIVIDING_VS_NORMAL_QUIESCENT_UP	183	4	3.64e-4	3.36e-2	DHFR, GGH, SHMT2, TYMS
BERENJENO_TRANSFORMED_BY_RHOA_UP	524	6	3.90e-4	3.36e-2	MTHFD2, DHFR, TYMS, DNMT1, AHCY, GART
ACEVEDO_NORMAL_TISSUE_ADJACENT_TO_LIVER_TUMOR_DN	338	5	3.91e-4	3.36e-2	GNMT, MAT1A, SHMT2, AHCY, CBS
SHAFFER_IRF4_TARGETS_IN_ACTIVATED_B_LYMPHOCYTE	77	3	3.94e-4	3.36e-2	CTH, AHCY, GART
WOO_LIVER_CANCER_RECURRENCE_DN	77	3	3.94e-4	3.36e-2	ALDH1L1, SARDH, MAT1A
SHEPARD_BMYB_MORPHOLINO_DN	195	4	4.63e-4	3.84e-2	DHFR, SARDH, DNMT1, TCN2
MELLMAN_TUT1_TARGETS_UP	18	2	5.03e-4	3.99e-2	MTHFD2, MTHFD2L
TERAMOTO_OPN_TARGETS_CLUSTER_7	18	2	5.03e-4	3.99e-2	CTH, MTHFD2
CEBALLOS_TARGETS_OF_TP53_AND_MYC_UP	19	2	5.62e-4	4.25e-2	CTH, MTHFD2
JEON_SMAD6_TARGETS_DN	19	2	5.62e-4	4.25e-2	CTH, DHFR
GRAHAM_NORMAL_QUIESCENT_VS_NORMAL_DIVIDING_DN	88	3	5.83e-4	4.27e-2	DHFR, GGH, TYMS
WONG_MITOCHONDRIA_GENE_MODULE	208	4	5.90e-4	4.27e-2	MTHFD2, CHDH, MTRR, SLC25A32
CAIRO_HEPATOBLASTOMA_CLASSES_UP	570	6	6.07e-4	4.31e-2	DNMT3A, ATIC, DNMT1, PRMT1, AHCY, GART
CAIRO_LIVER_DEVELOPMENT_DN	216	4	6.80e-4	4.72e-2	ALDH1L1, BHMT, GNMT, CBS
CROONQUIST_IL6_DEPRIVATION_DN	95	3	7.29e-4	4.96e-2	DHFR, GGH, TYMS
